# A review of artificial sebum formulations, their compositions, uses and physicochemical characteristics

**DOI:** 10.1111/ics.13022

**Published:** 2024-09-09

**Authors:** Nicole Rosik, Jon A. Preece, Peter J. Fryer, Ian McRobbie, Zhenyu J. Zhang

**Affiliations:** ^1^ School of Chemical Engineering University of Birmingham Edgbaston UK; ^2^ School of Chemistry University of Birmingham Edgbaston UK; ^3^ Innospec Ltd, Innospec Manufacturing Park Cheshire UK

## Abstract

Sebum is a complex mixture of skin lipids responsible for lubrication, moisture retention and skin protection from external factors such as bacteria and fungi. The physicochemical properties of natural sebum are not well understood and are not easily accessible. Artificial sebum is widely used for sebum‐related research such as dermal bioaccessibility, fingerprint production, dermatology, removal and sebum studies. It was found that the composition of artificial sebum affects the bioaccessibility of metals and drugs as well as the growth of some strains of bacteria. Squalene present in sebum was also found to be responsible for creating yellow stains on fabrics, whereas an increased concentration of fatty acids and triglycerides can lead to higher malodour of fabrics. Moreover, sebum and artificial sebum are poorly characterized with only 20 of 81 formulations characterized by certain techniques such as differential scanning calorimetry, nuclear magnetic resonance and thin‐layer chromatography. This article reviews the artificial sebum formulations reported in the open literature between 1965 and 2023. We have discussed the compositions, uses and characterization techniques of artificial sebum used in the previous work and compared their properties to those of human sebum. A total of 81 artificial sebum formulations were found across the literature with 17 new formulations identified. The artificial sebum composition varies greatly between publications and there is no consistent formulation. There is a wide range of chemicals that are used as the main components of artificial sebum. We have highlighted the effect of chemical composition and individual compounds on the overall properties of the artificial sebum reported, and recommend that there is a great potential for creating personalized cosmetics and home care products once the characteristics of sebum are better understood.

## Graphical abstract

We comprehensively reviewed the compositions and characterizations of artificial sebum reported in the open literature between 1965 and 2023, and compared their properties to those of human sebum.

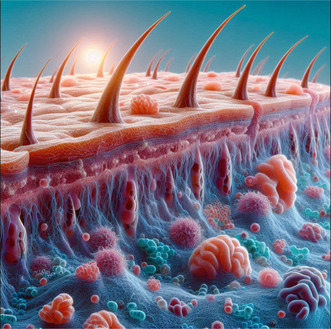

## Résumé

Le sébum est un mélange complexe de lipides cutanés responsable de la lubrification, de la rétention d'humidité et de la protection de la peau contre des facteurs externes tels que les bactéries et les champignons. Les propriétés physico‐chimiques du sébum naturel ne sont pas bien comprises et ne sont pas facilement disponibles. Le sébum artificiel est largement utilisé pour la recherche liée au sébum, comme la bioaccessibilité cutanée, la production d'empreintes digitales, la dermatologie, le retrait et les études sur le sébum. Il a été constaté que la composition du sébum artificiel affecte la bioaccessibilité des métaux et des médicaments ainsi que la croissance de certaines souches de bactéries. Le squalène présent dans le sébum s'est également avéré responsable de la formation de taches jaunes sur les tissus, tandis qu'une concentration accrue d'acides gras et de triglycérides peut entraîner une odeur désagréable plus élevée des tissus. En outre, le sébum et le sébum artificiel sont mal caractérisés avec seulement 20 formulations sur 81 caractérisées par certaines techniques telles que la calorimétrie à balayage différentiel, la résonance magnétique nucléaire et la chromatographie sur couche mince. Cet article examine les formulations de sébum artificiel rapportées dans la littérature ouverte entre 1965 et 2023. Nous avons discuté des compositions, des utilisations et des techniques de caractérisation du sébum artificiel utilisé dans l'ouvrage précédent et comparé leurs propriétés à celles du sébum humain. Au total, 81 formulations de sébum artificiel ont été trouvées dans la littérature, avec 17 nouvelles formulations identifiées. La composition du sébum artificiel varie considérablement d'une publication à l'autre et il n'existe pas de formulation cohérente. Il existe un large éventail de produits chimiques qui sont utilisés comme principaux composants du sébum artificiel. Nous avons souligné l'effet de la composition chimique et des composés individuels sur les propriétés globales du sébum artificiel rapporté, et nous soutenons qu'il existe un grand potentiel pour la création de produits cosmétiques et de soins à domicile personnalisés une fois que les caractéristiques du sébum seront mieux comprises.

## INTRODUCTION

**FIGURE 1:**
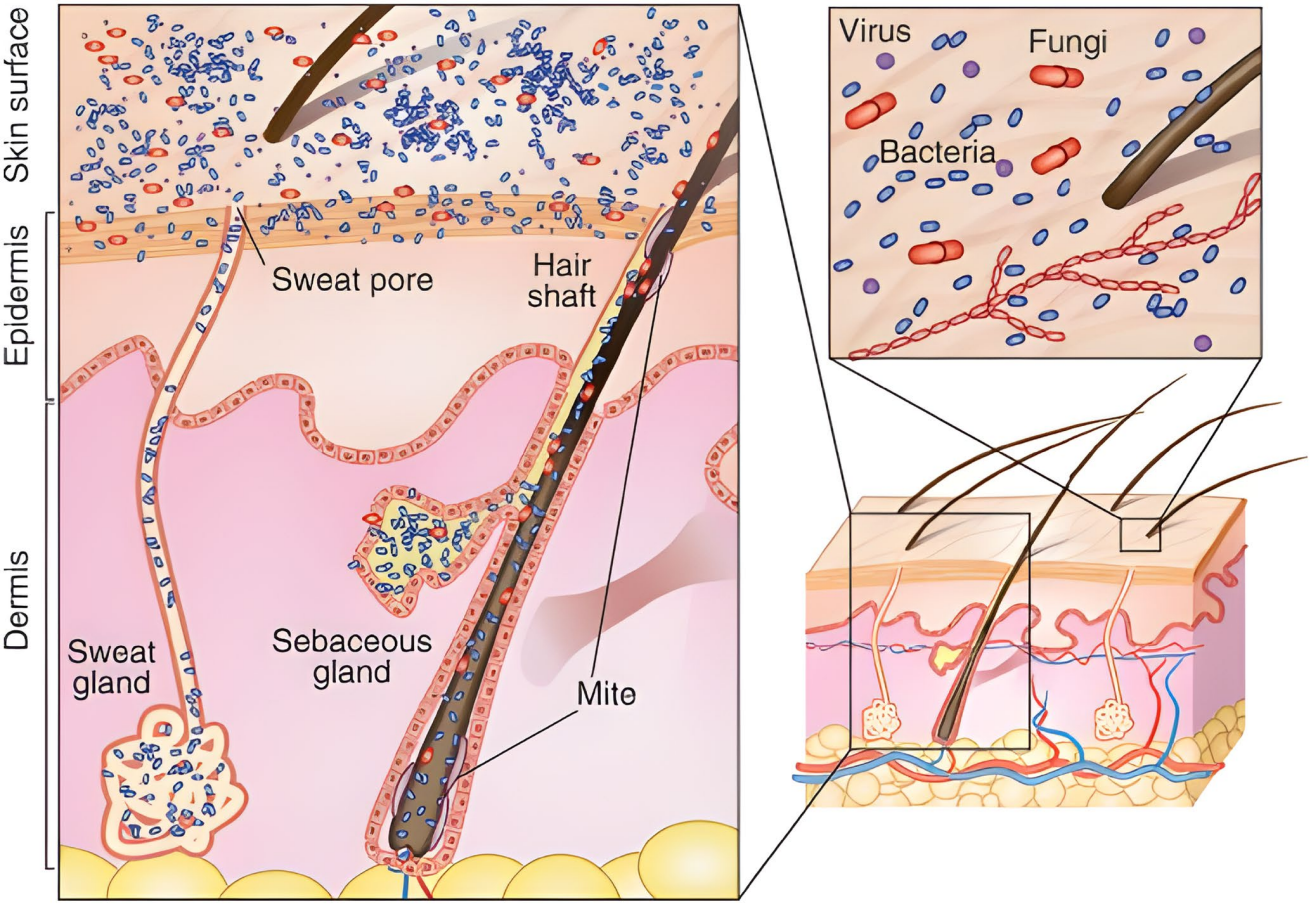
Schematic of skin cross‐section with representation of bacteria, viruses, fungi and mites covering the surface of the skin. Reproduced from Kong et al. [2] with permission from Elsevier.

Skin in its entirety is the first barrier to stop harm to the human body. It provides a physical barrier to the trauma of physical breaches from external objects, as well as a barrier to microorganisms to inhibit infection [1, 2]. However, the surface of the skin has to be extremely resilient against several factors, such as:

- mechanical force [3]
- chemical exposure including
  - deliberate exposure to chemicals, such as surfactants in soaps and volatile organic compounds present in perfumes, as well as cosmetics and topical medications, and
  - indirect exposure to chemicals, materials or nanomaterials on contact surfaces, in the air or aqueous solutions (pools, sea or rain)
- biological organisms, such as bacteria, viruses and fungi, that may also be present on contact surfaces and in the air [1] as presented in Figure 1
- ambient light, in particular, UV light.

To protect against these factors, the human skin has evolved sebaceous glands over the entire skin surface (apart from palms and soles), which secrete a complex mixture of lipids that:

- provide skin lubrication, by virtue of its waxy nature,
- maintain skin hydration, by reducing transepidermal water loss,
- provide a barrier to external chemicals, microorganisms and (nano)materials reaching the actual skin surface [1, 4, 5]

In addition, sebum is also an important part of the overall skin microbiome where it provides an environment in which ‘good’ microorganisms can thrive. Examples include *Staphylococcus Epidermidis*, which helps in wound healing, fighting against pathogens and immune system modulation [6], and *Propionibacterium acnes*, which hydrolyse triglycerides to form fatty acids, resulting in an acidic skin pH, which kills pathogens [1]. However, overproduction of those bacteria can also lead to negative effects such as atopic dermatitis caused by *S. epidermidis* [6] or acne caused by *P. acnes* [1].

Human sebum consists of a mixture of lipids including squalene, waxy esters, triglycerides, fatty acids, cholesterol esters and cholesterol [7, 8, 9]. A representative composition of human sebum is summarized in Table 1. The actual composition of the sebum of humans varies not only upon the point of production on the skin [8, 9, 10] but also on the age, gender, diet and underlying health conditions of an individual, as well as their geographical location and the climate in which they live [8]. Human sebum has been previously characterized by various methods: the most important physicochemical properties of human sebum are listed in Table 2 and are later compared to artificial sebum.

**TABLE 1:** Representative human sebum composition based on the reported studies [9, 11, 12, 13].

Component	Amount [wt%]
Triglycerides	31.3
Free Fatty Acids	25.1
Wax Esters	25.2
Squalene	13.6
Cholesterol Ester	2.6
Cholesterol	2.2

**TABLE 2:** Human sebum properties.

Property	Value
DSC	Sharp peaks at −25°C and 25°C [14] Melting points at 32.9°C and 42.0°C [15]
NMR	Peaks at: 5.04–5.13 ppm squalene, 4.20–4.30 ppm triglycerides, 3.96–3.99 wax esters [15] Peaks at: 5.06–5.13 ppm squalene, 4.52–4.60 ppm cholesterol esters, 4.21–4.30 ppm triglycerides and 3.96–3.99 wax esters [16]
TLC	TLC of sebum with the order of sebum components: free fatty acids (bottom), triglycerides (middle), wax esters, cholesterol esters and squalene (top). No Rf values were given; however, extract was analysed using GC‐FID [4]
Coeff. of Friction (CoF)	No information on CoF of sebum itself on skin; however, there are many publications on skin tribology, refer to Derler and Gerhardt [17]
Surface tension	25 dyn/cm at 30°C and room temperature [12, 18]
IR	1740 cm^−1^ (C – O fatty esters), 1710 cm^−1^ (C – O fatty acids) and 1460 cm^−1^ (CH_2_ hydrocarbons) [10]
Absorption	Absorption peaks at: 1212, 1364 nm (weak); 1720, 1760 (stronger); and 2306, 2346 nm (strong) [19]
Rheology	Viscosity 997.5 millipoises at 26.5°C and 551.9 millipoises at 38°C [12]

Given the importance of sebum to the skin surface, much research has been directed at how such exposures impact skin and are mitigated by the protective sebum layer. However, these studies have been focused on artificial skin and artificial sebum because human sebum is not easily accessible given that skin produces only 1 mg cm^−2^ of sebum every 3 h [20]. It is challenging to collect volumes of human sebum for experimental purposes, and so a significant amount of research has been directed at producing artificial sebum.

The first artificial sebum formulation was proposed in 1935 when Colin‐Russ evaluated human foot perspiration and its interactions with footwear, which inspired further research on this topic [21, 22, 23, 24, 25]. However, little was then known about the composition of human secretions due to the limited analytical techniques available. It was not until 1965 that Spangler et al. published the first paper detailing an artificial sebum composition which was similar to human sebum [26].

Stefaniak and Harvey [27] comprehensively reviewed, in 2006, artificial skin surface film liquids (SSFL) for dissolution studies and suggested an appropriate SSFL formulation, which was subsequently used by many researchers as a basis for artificial sebum formulations.

Since 1965, there have been at least 17 new formulations of artificial sebum suggested in the literature. However, there is a great degree of variation between the reported formulations, which could lead to inconsistency in laboratory testing, for example, in the dissolution studies of chemicals. Moreover, depending on the composition and presence of specific components of sebum, the artificial sebum will have different physicochemical and mechanical characteristics. Having a well‐characterized and consistent formulation of artificial sebum, which reproduces the properties of natural sebum, is thus critical for generating reliable and consistent results for academic and industrial research.

This review will build upon the 2006 review of Stefaniak and Harvey by comparing the 17 compositions of artificial sebum that have been reported not only for dissolution studies but also for cosmetic, forensic, dermatology and sebum analysis studies. In addition to the chemical composition, physicochemical characteristics of artificial sebum are reviewed critically, to provide those who use artificial sebum for academic and industrial research with an informed decision in choosing or preparing artificial sebum. Therefore, this review will:

- itemize the composition of artificial sebum formulations and compare them with human sebum,
- comprehensively review previous work (academic and industrial) using artificial sebum,
- evaluate the physicochemical and mechanical properties of artificial sebum and compare them with human sebum.

It is worth highlighting that the review is focused on artificial sebum exclusively, without coverage of other biological fluids, such as sweat, that were often considered when working on artificial sebum.

## METHODS

**FIGURE 2:**
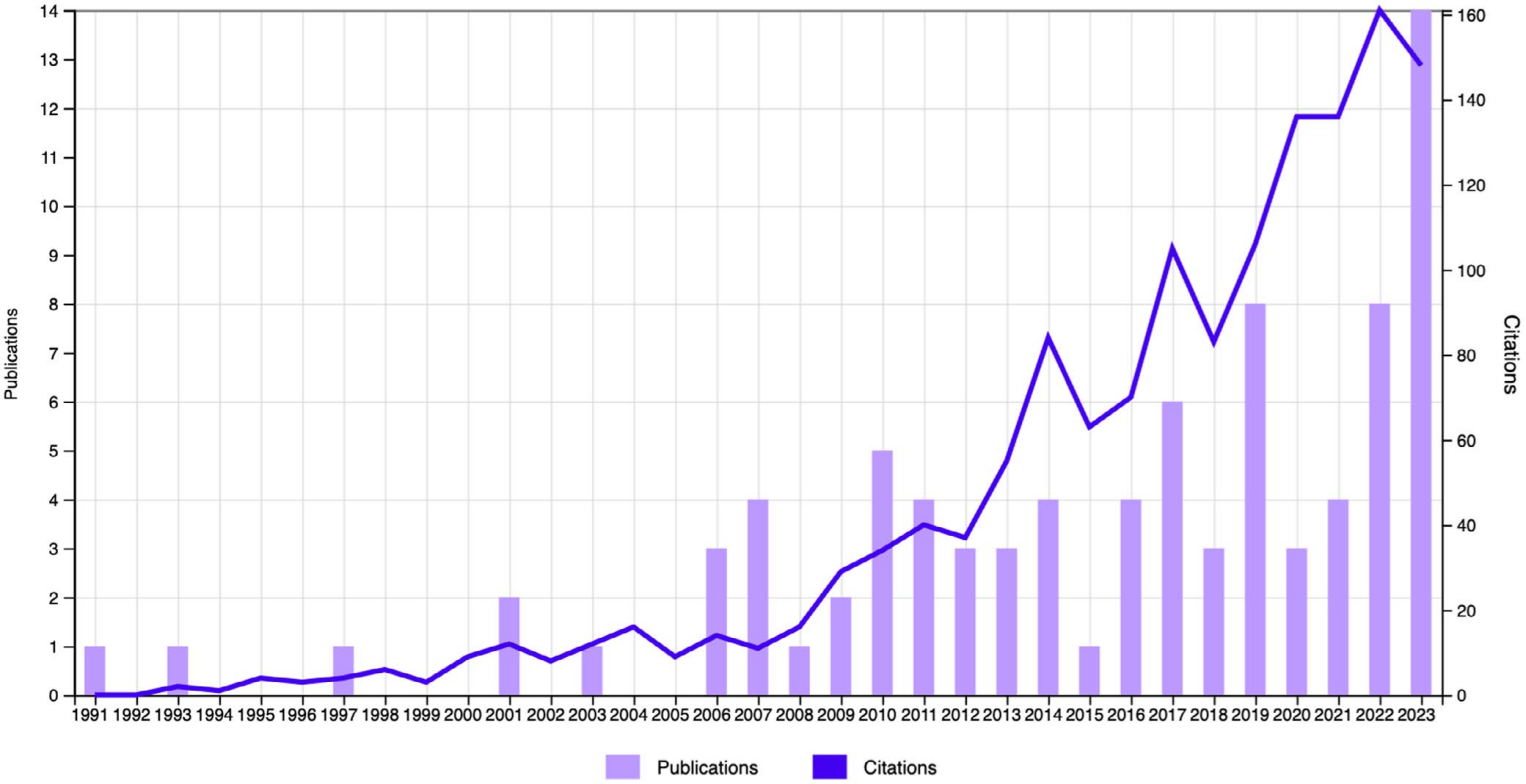
Web of Science citation report after searching for ‘artificial sebum’, 9th November 2023, https://www.webofscience.com.

The literature search was carried out using Google Scholar and Web of Science as the search engine for publications between 1965 and 2023. The keywords searched for were as follows: ‘artificial sebum’, ‘model sebum’ and ‘skin sebum’. As of 9th November 2023, the phrase ‘artificial sebum’ yields 86 publications, while the other two yield 304 and 1,811 results respectively. The latter two keywords identify publications that are related to skin, acne and sebum secretion, and thus do not use artificial sebum formulations, and are beyond the scope of this review. Of the 86 publications concerning artificial sebum, only 75 were relevant for this review. The increasing number of publications and citations (Figure 2) each year evidences an increased interest in the investigation and usage of artificial sebum. Despite this, the overall number of publications on this topic remains limited.

In total, 81 artificial sebum formulations have been reported in the literature. Some papers that mention ‘artificial sebum’ used the words ‘artificial’ and ‘sebum’ separately, so the actual number of formulations was less than the number of returns from the Web of Science search.

The relevant publications are summarized and categorized by year, novelty of the formulation (new or based on previous research), chemical composition of the artificial sebum used and the characterization methods, in Table S1.

## CHEMICAL COMPOSITION OF ARTIFICIAL SEBUM

The chemical composition of artificial sebum was categorized by the components used. Seventeen new artificial sebum formulations have been identified in the literature. In these papers, the authors did not cite any existing protocols for creating artificial sebum. Table 3 presents all these ‘new formulations’ identified.

**TABLE 3:** New artificial sebum formulations reported in the literature.

Author	Year	Squalene	Triglycerides	Wax esters	Free fatty acids	Cholesterol	Cholesterol esters	Other
Spangler et al. [26]	1965	✓	✓	✓	✓	✓		
Blanc et al. [29]	1989	✓	✓	✓	✓		✓	
Nelson et al. [38]	1993	✓	✓		✓	✓		✓ Octadecanol
Skopp et al. [39]	1997	✓	✓	✓	✓	✓		
Mainkar and Jolly [40]	2000		✓	✓	✓			
Musial and Kubis [41]	2003	✓	✓	✓	✓	✓		
Katsuta et al. [42]	2005		✓		✓			
Stefaniak and Harvey [27]	2006	✓	✓	✓	✓	✓	✓	
Mo et al. [18]	2007	✓	✓	✓	✓	✓	✓	
Wertz [30]	2009	✓	✓	✓	✓			✓ Vitamin E
Yokoi et al. [35]	2014	✓	✓	✓	✓	✓	✓	✓ Carbon Black
Sisco et al. [43]	2015	✓	✓	✓	✓	✓	✓	
Galliano et al. [44]	2017				✓			✓ Thermal water
Peterson et al. [36]	2017	✓	✓	✓	✓	✓		✓ Water, carbon black, iron oxides, preservatives
De la Hunty [34]	2017	✓	✓		✓	✓	✓	✓ Vitamin E
Spittaels and Coenye [45]	2018	✓	✓	✓	✓			✓ Vitamin E
Suzuki et al. [46]	2022	✓	✓		✓			✓ Diglycerides

Most formulations contained triglycerides, wax esters and free fatty acids. Squalene, even though it has been shown [28] to have a significant effect on the physicochemical properties of sebum, was not always added to the formulation. A few authors [18, 27, 29] included cholesterol esters in their formulations to more closely resemble human sebum. In addition to the basic formulation, vitamin E has often been added to the formulation as it is also present in human sebum [30, 31, 32, 33, 34]. Depending on the intended use of the artificial sebum, ingredients such as carbon black [35, 36, 37] and iron oxides can be added as surrogates of air pollutants [36, 37], as well as preservatives to maintain the stability of sebum formulation [36, 37].

Based on these formulations, many researchers have adjusted the artificial sebum formulations to their needs, for example, the components of each formulation and their ratios. The composition of each formulation is similar, as it consists of components from the same ‘family’ of compounds. However, various chemicals are used such as triglycerides, wax esters, free fatty acids and cholesterol esters. Table 4 summarizes the compounds that have been used to act as unsaturated and saturated triglycerides, wax esters, fatty acids and other components used in artificial sebum formulations.

**TABLE 4:** Chemicals used for formulating artificial sebum.

	Triglycerides	Wax esters	Free fatty acids
Unsaturated	Triolein Trimyristolein Tripalmitolein	Oleyl oleate Glyceryl trioleate	Oleic acid Palmitoleic acid Myristoleic acid Cacao butter Linoleic acid
Saturated	Trimyristin Tripalmitin Tristearin Tricaprin Tricaprylin Trilaurin Pork lard Olive oil Cotton seed oil Coconut oil Petrolatum Vegetable oil	Myristyl myristate Myristyl palmitate Palmityl palmitate Cetyl palmitate Stearyl palmitate Palmityl oleate Polyglyceryl oleate Stearyl stearate Wool wax Liquid paraffin Spermacetti wax Jojoba oil Glycerol triisostearate Isostearyl isostearate	Palmitic acid Stearic acid Myristic acid Lauric acid Cacao butter Hexanoic acid Heptanoic acid Octanoic acid Nonanonic acid Dodecanoic acid Tridecanoic acid Pentadecanoic acid Arachidic acid Hydrogenated palm oil

	Cholesterol esters	Cholesterol	Squalene
	Cholesterol Oleate Cholesteryl Palmitate Stigmasterol	Cholesterol	Squalene Lanolin

Wax esters, triglycerides and free fatty acids make up the majority of artificial sebum. Of the 81 artificial sebum formulations, wax esters were included in 65 formulations, triglycerides in 74, free fatty acids in 72, squalene in 66, cholesterol esters in 38, cholesterol in 55 and other components in 20.

**FIGURE 3:**
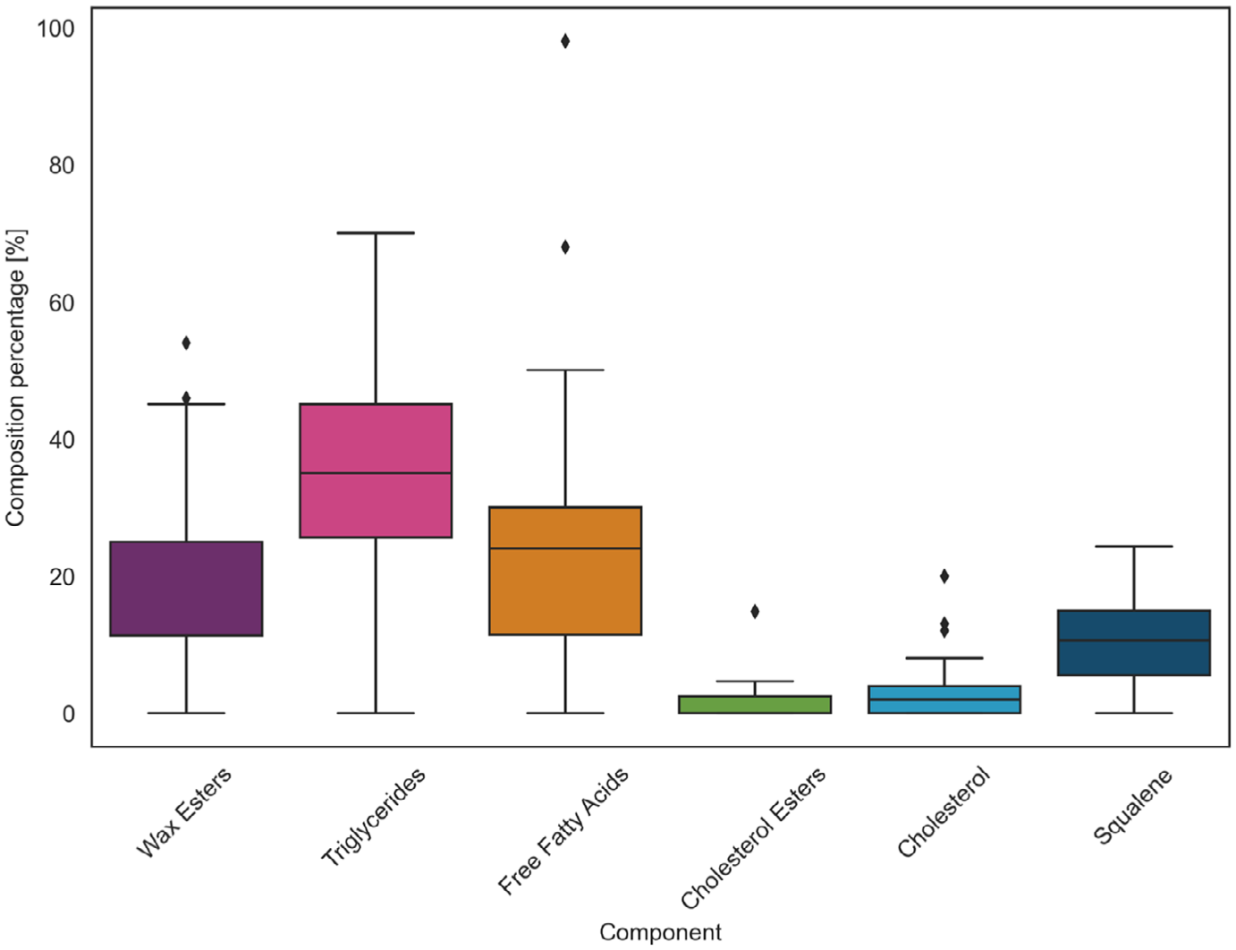
Composition of wax esters, triglycerides, free fatty acids, cholesterol esters, cholesterol and squalene in artificial sebum formulations.

The averaged fraction of each component in artificial sebum formulations is shown in Figure 3. The error bars in the box plot indicate confidence intervals for the concentration of the specific component used, the line in the middle of the box indicates the median of the concentrations used and the diamonds represent the outliers. Triglycerides take up on average 32.4 wt%, free fatty acids 25.4 wt%, wax esters 19.7 wt%, squalene 9.8 wt%, cholesterol 2.7 wt% and cholesterol esters 1.5 wt%.

Human sebum contains on average 31.3 wt% of triglycerides, free fatty acids 25.1 wt%, wax esters 25.2 wt%, squalene 13.6 wt%, cholesterol esters 2.6 wt% and cholesterol 2.2 wt% [9, 11, 12, 13].

Stefaniak and Harvey [27] performed similar composition analysis, in which they calculated the average amount of each artificial sebum component and compared them to human sebum. Based on the composition analysis, they suggested a standardized composition (Table 5) which could be used for laboratory testing for bioaccessibility studies.

**TABLE 5:** Composition of model sebum proposed by Stefaniak and Harvey [27].

Component	Amount [wt%]
Squalene	10.6
Wax esters	25.0
Triglycerides	33.0
Free fatty acids	28.3
Cholesterol esters	2.0
Free cholesterol	4.0

There are two standard methods of preparing artificial sebum. The first one adds all the ingredients of the artificial sebum to a glass beaker which is heated to 60°C while mixing until a clear solution is formed. The artificial sebum is then let to cool down [15, 26, 36, 47]. The other technique involves dissolving lipids in chloroform and methanol mixture at 2:1 ratio at an elevated temperature (usually 45°C). After mixing all the lipids, the solvent is evaporated [30, 48, 49]. Both methods require elevated temperature and mixing. The latter requires organic solvents, which allows better dissolution of the lipids and potentially provide a more homogeneous formulation.

## PUBLICATIONS INVOLVING ARTIFICIAL SEBUM

**FIGURE 4:**
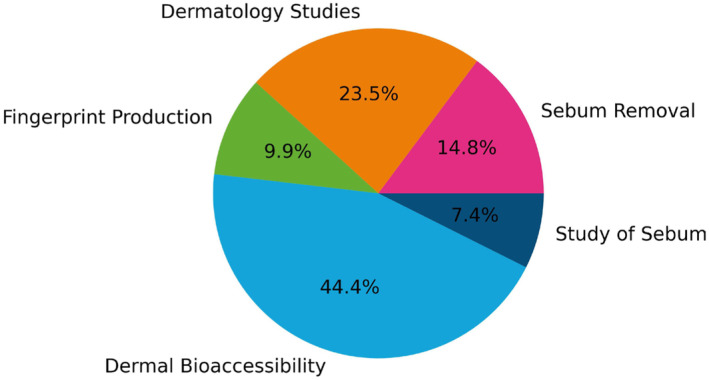
Categories of artificial sebum formulations used in various research areas based on 81 formulations (details of formulations and publications in Table S1).

A total of 81 artificial sebum formulations have been identified among the 75 publications recorded since 1965. The application of artificial sebum can be divided into five categories: (i) dermal bioaccessibility, (ii) fingerprint fabrication, (iii) dermatology studies, (iv) sebum removal and (v) the study of sebum itself. Figure 4 shows the percentage of each application in the open literature.

The largest fraction of the formulations reported (44.4%) was used for dermal bioaccessibility studies of various compounds, such as metal particles, drugs and compounds from indoor dust or cosmetics [27, 50, 51]. These studies focused on the migration of chemical compounds into sebum and the stratum corneum (the outer layer of the skin), for example, by measuring their partition coefficient.

The second largest area of interest in the use of artificial sebum is in dermatology studies (23.5%), which include the growth of bacteria, primarily *P. acnes* and therefore acne treatment [45, 52, 53].

A notable number (14.8 %) of artificial sebum publications focus on sebum removal from fabrics and evaluating the cleansing performance of surfactants and cleansing devices such as sonic brushes [36]. Artificial sebum has also been used for fingerprint fabrication for forensic tests [54] or to study anti‐fingerprint coating [32] and this takes up 9.9% of the publications.

Investigating the properties of sebum is critical for the understanding of the above‐mentioned research areas. However, only a small proportion of the publications (7.4%) were dedicated to the physicochemical properties of sebum. Preparation of new artificial sebum formulations, skin mimics or measuring tribological properties are topics covered in this area [48, 55, 56].

### Dermal bioaccessibility of chemicals

Penetration of metal particles, drug molecules, cosmetics and pollutants from air into skin and sebum is a research topic in which artificial sebum and sweat formulations are used extensively both to build ex vivo models and to develop fundamental understanding. Stefaniak and Harvey [27] reviewed the use of artificial sebum and sweat for dissolution studies of chemical constituents from various materials like jewellery, textiles, cosmetics or drugs. They concluded that many formulations were not similar to human sebum and sweat, and they suggested an improved SSFL to address the issue. In addition, it was found that there was no standardized dissolution test, so a dissolution test was suggested that controlled experimental temperature, storage, friction and characterization of the test materials [27].

A more recent review by Champmartin et al. in 2022 discussed the importance of selecting a suitable material to solubilize chemicals and highlighted the challenges in preparing a realistic surrogate for such absorption measurements [5]. They reviewed some commonly used solvents (water, ethanol and acetone) and complex mixtures (artificial sweat and artificial sebum) for dissolution studies. In their review, Champmartin et al. noted both that there was no standardized artificial sebum formulation and the amount of artificial sebum applied in skin tests was much greater than the actual amount of sebum on the skin. This lack of consensus in artificial sebum formulation and varying quantity implies that caution is needed when performing dissolution experiments. It is also worth noting that the authors discussed the effects of the ingredients present in artificial sebum [5].

One of the most common penetration studies using artificial sebum is transfollicular and transdermal penetration. Here, the absorption of chemicals or drug molecules occurs either through hair follicles and sebaceous glands or through the stratum corneum respectively [47]. Penetration studies are of great interest in assessing the risks of the potential exposure of skin and hair to agrochemicals and environmental pollutants [5, 57, 58, 59], drugs or metals from jewellery, soils or commonly used surfaces [33, 60]. Testing the penetration of drugs in vivo, ex vivo and in vitro is costly and time consuming, which is why artificial sebum is often used to provide a practical model system [57]. The transdermal penetration profile of drug molecules is fundamental for topical administration as it is non‐invasive, painless and offers targeted delivery [58, 61]. In all cases concerning migration of small molecules in a continuous medium, the physicochemical properties of the artificial sebum (e.g. viscosity and interaction with the penetrating molecules), which are determined by its composition, will have a critical impact on the results of ex vitro measurements.

The main categories of penetration studies use metals, drugs and chemicals found in cosmetics together with everyday use products such as plastics. Metals used in ex vivo transdermal studies include Ni [33, 60], Ca [42], Be [62], Cr [33], Pb [33] and Zn [33]. Hemingway and Molokhia (1987) used artificial sweat with small amounts of triglycerides to resemble sebum when studying the dissolution of metallic nickel [60]. In 2011, Stefaniak et al. used artificial sebum in a chamber used for dissolution of beryllium‐containing materials such as hydroxides, oxides or alloys in artificial sweat [62]. In that study, Stefaniak did not evaluate the effect of artificial sebum composition in the dissolution of beryllium‐containing materials, which was assumed to have no influence. They used a previously reported artificial sebum with a composition resembling human sebum [49]. However, Villegas and colleagues (2019) later demonstrated that the composition of artificial sebum could affect the dissolution of metals such as Cr and Zn [33]. It was found that dermal bioaccessibility of metals was low in two artificial sebum formulations which were based on the work of Stefaniak and Wertz [30, 49]. Furthermore, Marin Villegas and Zagury (2021) studied the dissolution of metal(loid)s from contaminated soils and found that the presence of sebum affects the permeation of metal(loids), specifically copper, by increasing the lag times of permeation [63]. Bioaccessibility of metal(loids) was recently investigated by Ghislain and Zagury (2023), who found that the presence of sebum in SSFL affects dermal bioaccessibility of As, Cu and Cr. [64]. In addition, the effect of sebum on the bioaccessibility in SSFL is dependent on the metal, soil and artificial sweat formulation [64]. It can be concluded that there is a difference in bioaccessibility of metals depending on the artificial sebum used [33, 63, 64], which suggests that a more standardized and representative formulation needs to be used.

Apart from permeation of metal(loid)s, another large area of research is dermal bioaccessibility of drugs. Motwani et al. initiated research into that area in 2001 by investigating which drug delivery vehicles are miscible with sebum with the use of differential scanning calorimetry (DSC). They hypothesized that the more miscible the vehicle is, the more effective it will be for drug delivery [65]. They found that DSC is a good testing method for determining which vehicles are more miscible with sebum [65].

Valiveti, Lu and colleagues carried out extensive studies on drug penetration [15, 47, 66], including ketoconazole, minoxidil, ibuprofen or salicylic acid [47, 66, 67, 68]. Their studies differed from others in their characterization of artificial sebum with DSC and NMR to ensure that the properties of artificial sebum were similar to those of human sebum [47, 66, 67, 68]. The main objective of their studies was to understand the thermodynamic and kinetic properties of drug diffusion in artificial sebum. It was found that the molecules of interest were transported better in artificial sebum/water mixture than in octanol/water as sebum/water mixture is more sensitive to the chemical structure of drugs [66]. It was also found that the molecular structure of the drugs tested affects their diffusion. Charged molecules were found to move slower in sebum than neutral ones while increasing the molecular weight reduced the diffusivity [47]. The authors, however, did not discuss the mechanism of transport of charged molecules. The interaction of molecules with sebum affects their thermodynamic and transport kinetic properties, which suggests that formulating an artificial sebum closely resembling human sebum is critical for drug penetration studies.

Other drugs that have been studied are common illegal drugs (such as methamphetamine, amphetamine, 3,4‐methylenedioxymethamphetamine (MDMA) or cocaine) that can be found on surfaces [31, 39, 69]. In 1997, Skopp et al. [39] investigated the drug uptake into the hair fibre. They evaluated bleached hair that was exposed to artificial sebum and sweat which contained drugs like cocaine, morphine or codeine, and found a minimal uptake of drugs by sebum. However, the authors did not find an explanation for various uptakes in sebum due to the intermolecular interactions between drugs and sebum [39]. Parker and Morrison [31] found that the variation in the drug partition coefficient depends on the sebum composition and, more specifically, on the presence of fatty acids. Doran and Howitt [69] suggested that sebum presence on work surfaces could increase the longevity of drugs by limiting their evaporation and degradation. However, the drugs are more likely to be absorbed in the bloodstream through skin or be ingested as a result of an uptake from the surfaces. The molecular interaction between drugs and sebum underpins such critical studies.

A range of chemicals have been studied using artificial sebum and include alcoholamines [41, 70, 71], caffeine [57, 67], silicone elastomers [72, 73, 74], pesticides [38, 75], fluorescent dyes [76], plasticizers from polyvinyl chloride (PVC) products [77, 78], bisphenols A and S [79], coacervate salts [80, 81], retinoid active pharmaceutical ingredients [82], ethanol and toluene [59], peptides [83], organic flame retardants or polycyclic aromatic hydrocarbons from indoor dust [50, 51].

Combined dermal bioaccessibility of organic flame retardants from indoor dust and the effect of cosmetics applied on the skin was examined by Pawar et al. (2017), who found that dermal bioaccessibility changes depending on the cosmetic applied [50]. For instance, sunscreen lotion could enhance bioaccessibility, whereas body spray decreased it [50]. They postulated that various properties of the cosmetics formulation factors, such as ionic strength, lipid content and contact time, could affect the bioaccessibility of chemicals from indoor dust [50].

In general, the bioaccessibility of various compounds depends on the molecular interactions with the sebum used, which thus requires fundamental understanding of the sebum. Studies so far show that the composition of artificial sebum affects the partition coefficients of molecules. This highlights the importance of having a standardized and well‐characterized artificial sebum that has the same behaviour as the real material for use in testing.

### Dermatology studies

Cosmetic and dermatological studies refer to the research work that considers the development of cosmetics, skin care products and skin surface microbes.

### Bacterial growth in artificial sebum

Artificial sebum is often used as a medium for growing bacteria. One of the most common bacteria that causes skin problems is *Propionibacterium acnes* (more recently referred to as *Cutibacterium acnes*) [45, 84]. Studies of bacteria or fungi that grow on the skin form most of the dermatology‐related sebum studies. Pannu et al. (2011) explored the antibacterial effect of nanoparticle‐based therapeutics against *P. acnes* and found that the synergy between nanoemulsions results in an effective acne treatment [52]. The influence of artificial sebum was also considered. Spittaels and Coenye, in a separate work, developed an artificial sebum formulation that can sustain the growth of *P. acne* for a week [45]. Biofilm preparation for growth of cutaneous bacteria using artificial sebum was also considered by Mart'Yanov et al. (2022). However, they used a simplified artificial sebum formulation that consisted of two ingredients, glycerol tripalmitate and jojoba oil. The artificial sebum was then mixed with keratine and agarose to form pellets which were further used for biofilm cultivation [85]. They reported that all the strains of bacteria tested could grow on the pellets using keratine and fatty acids as their carbon source. Based on these findings [85], they claim that the newly developed method is well suited for cultivation of biofilms, but note that a more complex artificial sebum should be used to make studies more representative. Suzuki et al. (2022) investigated the growth of *Malassezia* bacteria on human skin in sebum containing a modified Leeming and Norman agar medium (mLNA) and found that bacteria grew better in sebum containing mLNA rather than standard mLNA alone [46].

Another synthetic skin‐like growth media were studied by Swaney et al. (2023) who found that different bacteria grow differently [86] depending on the concentration of artificial sweat and sebum. It was found that some bacterial strains that are found on human skin have low, high or no preference for growth in sebum. *Corynebacterium* and *Dietzia* genera have high preference for sebum, whereas *Staphylococcus aureus* showed little growth at high concentrations of sebum and sweat [86]. The presence of free fatty acids in sebum is known to have antimicrobial effects and therefore many strains do not grow well in sebum [86]. Borrell and co‐workers (2019) studied how various strains of *P. acnes* adapt to the surrounding environments, one of which was a sebum‐like medium. They found that the acneic strain of *P. acnes* prefers growth in a lipid‐rich environment, like sebum, leading to enhanced acne [84].

Acne treatment using hydrogels with an antimicrobial ingredient (tetracycline) was studied by Kostrzebska et al. (2023) who evaluated the activity of alcoholamines contained in the hydrogels against model sebum [53]. However, it was previously found that stearic acid reacts with alcoholamine, resulting in saponification [41, 70, 71]. Kostrzebska's study found that the stearic acid from the artificial sebum embedded into a hydrogel did not react with alcoholamines through saponification [53]. This suggests that various components will react differently depending on the nature of the sebum environment.

Artificial sebum has also been used in studies such as the expression of surfactant proteins in human skin [18], the use of microemulsions for cosmetics and sebum stabilization [87], laser hair removal [19] and the evaluation of disinfectant wipes [88].

### Particle adhesion

**FIGURE 5:**
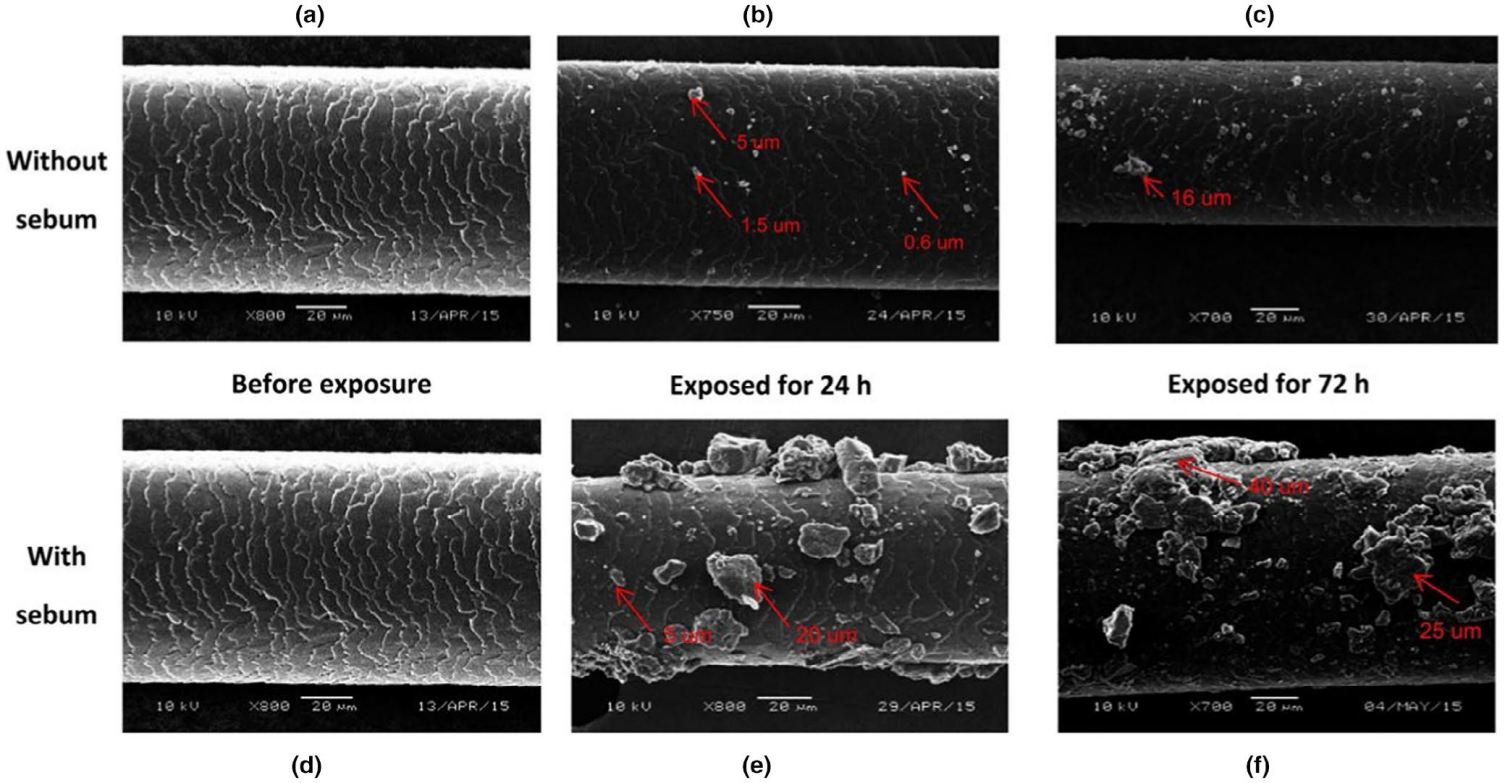
SEM images of hair swatches covered without (a) and with (d) artificial sebum, following exposure to aerial pollution for 24 h (b, e) and 72 h (c, f). Reprinted from Galliano et al. [44]. Copyright 2017 Society of Cosmetic Scientists and the Soci'et'e Fran¸caise de Cosḿetologie.

Dermatology studies have also involved the evaluation of particle adhesion to skin and hair. Galliano et al. (2017) reported that particles adhere more easily to sebum‐covered hair swatches than clean swatches, which then results in a loss of shine and increased friction (Figure 5) [44]. In addition, Stefaniak et al. (2021) measured particle adhesion to cotton gloves covered in artificial sebum and found that the higher the sebum levels on the material, the lower the adhesion of particles [89]. Particle adhesion to skin and sebum‐covered materials is dependent on factors such as molecular interactions, capillary condensation or electrostatic forces, none of which is fully understood [89].

The choice of artificial sebum will influence particle adhesion because of the different intermolecular interactions between the sebum ingredients and the particles, which again highlights the importance of having a consistent artificial sebum formulation.

### Sebum removal

The need for cleansing and removal of sebum underpins the performance of personal care products. Sebum removal is in all cases dependent on the surface energy of the substrate that sebum is deposited on, whether it is fabric, hair or skin.

Previous publications have focused on the removal of sebum from fabrics or skin. For example, Spangler et al. (1965) used an artificial sebum combined with a particulate mix and applied it on cloth to determine factors that influence detergency and to develop a reproducible testing technique [26]. Building on Spangler's work, Thompson and co‐workers developed a suite of reproducible soiling processes and detergency evaluation protocols [90]. One of the difficulties of detergency testing lies in the choice of soil and substrate [90]. A similar challenge was reported by Mainkar and Jolly (2000), who claimed there was no standard for the evaluation of shampoos and proposed a standardized method [40]. Around that time, the American Society of Testing and Materials released a standard for artificial sebum (ASTM D4265), which has been subsequently used by some researchers and has become the industrial standard [91, 92, 93]. Peterson et al. [36] and Galliano et al. [37] developed a sebum pollution model for use in evaluating cleaning formulas and methods, which comprises sebum mixed with air pollutants that have a negative effect on skin health. In all the soiling and cleaning tests, sebum has been mixed with various pollutants. However, none of the tests has considered the interaction between sebum and pollutants. Addition of pollutants could change the surface energy of sebum and lead to different test results.

Understanding the interactions among the soil, detergent and substrate will offer the fundamental knowledge underpinning cleansing technologies. Sebum is a complex soil that consists of both polar and non‐polar components. Thompson et al. [90] reported that polar sebum components are more readily removed than non‐polar components, and that the degree of removal of non‐polar components of sebum was determined by the surfactant used.

**FIGURE 6:**
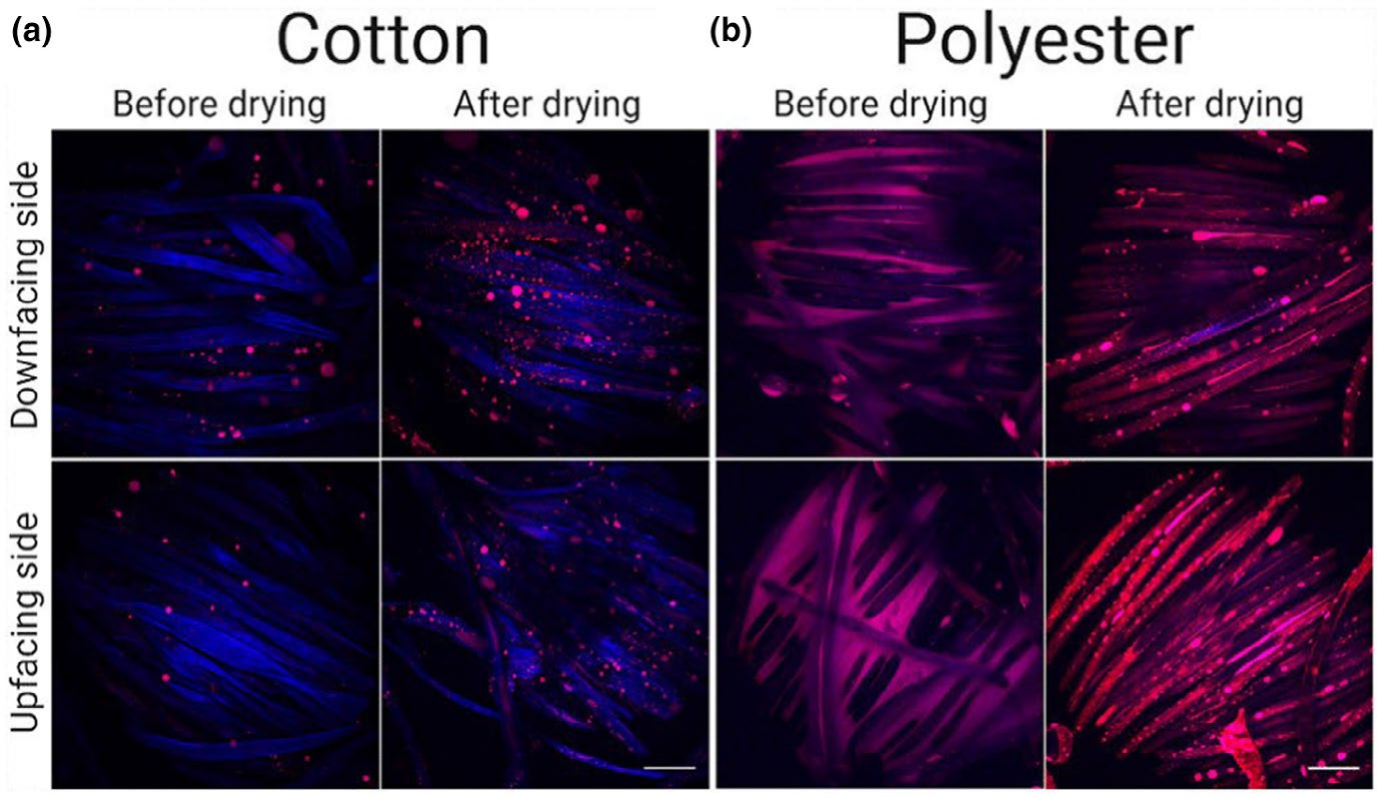
Distribution of artificial sebum stained with Nile red on cotton (a) and polyester (b) before and after drying on two sides of the fabrics. Fibres autofluorescence in the blue region of the spectrum. Reprinted from Møllebjerg et al. [95]. Open access article under the Creative Commons Attribution 4.0 International licence.

Another challenge faced by many consumers is the removal of old stains and malodour from garments that are in contact with skin. A number of tests using artificial sebum and dirt have been carried out. Bad odour and discolouration of fabrics are caused both by oxidation of sebum and by colonization and growth of skin bacteria [94, 95]. Squalene, one of the major components of sebum, is more easily removed when present alone compared to when it is mixed in sebum [91]. Oxidation of squalene produces some polar components that can react with the other compounds in sebum, resulting in high‐molecular‐weight products that are more difficult to remove [91]. Squalene is also the compound responsible for yellow stains on clothes [91]. On the other hand, the degradation of fatty acids and triglycerides results in the formation of short fatty acids that lead to malodour [95]. Due to its non‐polar characteristics, sebum has a higher preference for deposition on hydrophobic textiles such as polyester than cotton, which results in stronger odours and more discolouration on those fabrics [95]. Figure 6 shows the distribution of stained artificial sebum on cotton and polyester before and after drying, which shows the greater amounts of sebum on synthetic fabrics due to the preferred surface interaction.

The effect of lipase on stain removal from various fabrics was evaluated by Varanasi et al. [92] who found that lipase enhances sebum removal. It is well known that surfactants remove sebum. However, the challenge is that complete removal of sebum from the skin leads to irritation. Yokoi et al. [35] have investigated this and developed a surfactant system that removes sebum better than a reference cleanser, but that does not penetrate into the stratum corneum.

Overall, sebum removal is a very important aspect of the fast moving consumer goods (FMCG) industries and is slowly being better understood. However, the interactions among sebum, surfaces and detergents are not fully understood.

### Fingerprint production

Fingerprints usually consist of eccrine and sebaceous secretions, differ between individuals and depend on external factors, such as contact with cosmetics and food [11, 43, 96]. Fingerprint scientists deal with the challenging task of identifying various components in the fingerprint, which usually include sweat, sebum, cosmetics, food or drugs. However, fingerprints are susceptible to alterations through both chemical and physical changes over time [11, 43, 97, 98].

This inherent variability poses a considerable challenge for scientists aiming to identify the chemicals present in fingerprints. Consequently, researchers are actively engaged in developing test materials that mimic the actual human fingerprints [11, 43, 99], which has the added benefit of minimizing the risk of exposure to hazardous substances like explosives and narcotics if real prints were used [43].

Preparing natural fingermark secretions is challenging due to the variability between fingermarks [11, 97]. Children's secretions tend to disappear more quickly from a solid substrate due to the presence of mainly aqueous compounds and fewer organic compounds than that of adults [97, 100]. Moreover, the presence of squalene and cholesterol can change the molecular interactions between the components of sebum due to the oxidation and decomposition of the molecules [100]. Significant research has gone into formulating a realistic fingermark [43, 96, 98] and developing detection techniques using a physical developer [34]. Even though artificial fingermarks can be produced in such a way that the physical properties are similar, the detection technique using a physical developer does not react with artificial and natural sebum in the same way [34, 96].

Adhesion of fingermarks to a solid substrate depends on the surface interactions. The more porous the surface is, the more adhesion is observed, with penetration of up to 60 *μ*m [97]. Surface interactions are crucial when developing easy‐to‐clean surfaces such as electronics or household materials [101]. According to Stoehr et al. (2016) and Girod et al. (2012), the higher the temperature, the easier the removal of fingerprints, which is because at higher temperatures sebum melts leading to lower surface interactions with an ease of surface removal [97, 101]. An easy‐to‐clean surface could also be amphiphobic with low surface energies to reduce the intermolecular attractive forces and therefore reduce adhesion [32]. Druart and colleagues [32] developed an anti‐fingerprint glass coating using enzymes, which causes degradation of sebum and therefore disappearance of the fingerprint from the surface after 3 days.

### Study of sebum

The complexity of sebum makes understanding its physical and biological properties an important and interesting area of research. The first publication on the analysis of human sebum dates back to 1949 when Butcher and Coonin measured the physical properties of human sebum [12]. This was followed by investigating the chemical composition and the variability of sebum between individuals, which gave researchers some great idea on how to create an artificial sebum [9, 13, 102]. Based on this, many researchers have started developing their own formulations based on their needs. One of the most widely used formulations was developed by Wertz, Stefaniak and Harvey, who not only suggested a new artificial sebum formulation but measured its stability [30, 49, 103].

In 2001, Motwani et al. used differential scanning calorimetry to study model sebum formulations and evaluated the effect of each component of sebum on the melting point. This is discussed in more detail in the DSC Section. Later the tribological effect of sebum was evaluated by Bhuyan et al. (2006), Gerhardt et al. (2009) and Korbeld et al. (2020), which is reviewed in the Tribology Section [28, 48, 56].

## PHYSICAL, CHEMICAL AND MECHANICAL PROPERTIES OF SEBUM

A total of 81 artificial sebum formulations were reported in the literature, of which only 20 were characterized. Of the 17 new formulations suggested in the literature, only 4 have been characterized using surface tension, Thin‐layer chromatography (TLC), secondary ion mass spectrometry (SIMS), stability and tribology [18, 30, 43, 44, 100]. Other techniques that have been used to investigate artificial sebum are differential scanning calorimetry (DSC), contact angle (CA), nuclear magnetic resonance (NMR), optical microscopy, infrared spectroscopy, absorption measurements, scanning electron microscopy (SEM), rheology and molecular dynamics.

### Thermal properties

The most common method of characterization of artificial sebum is DSC, which allows the determination of melting points [14]. Motwani, Rhein and Zatz found four melting points of model sebum, at −20°C, 15°C, 40°C and 55°C [55]. They concluded that the model sebum is crystalline in nature and the presence of multiple melting peaks suggests the presence of multiple phases. Human skin temperature is around 33°C; sebum will be a mixture of phases at that temperature and specific components of sebum are not miscible with each other [55]. In another study, Motwani, Rhein and Zatz [65] placed various vehicles for drug delivery into the same tested artificial sebum to check miscibility and the effect on the thermal transitions. They concluded that depending on the vehicles added, the higher melting points, that is, 40°C and 55°C, were affected. It was found that hydrophobic materials could reduce the melting point at 40°C, whereas hydrophilic compounds had no effect on that peak. The addition of drug delivery vehicles had little effect on the negative melting points [65].

Valiveti et al. [47, 66] characterized an artificial sebum formulation using DSC and reported the major transition points to be 30.63°C and 36.07°C. It is surprising that only two melting points were found here as the DSC measurement was carried out between −40 and 60°C under the same conditions that Motwani, Rhein and Zatz found four melting points [55]. The chemical difference between the two studies was that cholesterol and cholesterol esters were present in the Valiveti sebum but not in Motwani's. The peaks observed below 0°C were due to unsaturated compounds in sebum such as tripalmitolein and palmitoleic acid.

Lu et al. compared four artificial sebum formulations with sebum extracted from humans and hamsters, using DSC as one of the primary characterization methods [15]. The artificial sebum formulations were prepared based on those of Nordstrom et al (1986), Spangler et al. (1967), Friberg and Osborne (1986) and the last one was prepared by Lu and colleagues [7, 15, 104, 105]. Again, the transition peaks identified were not sharp due to the presence of waxy materials. The melting points of artificial sebum L (prepared by Lu et al.) were 33.3°C and 40.28°C which are close to the human sebum melting points at 32.9°C and 42°C. The artificial sebum formulation developed in this publication has been used by many other authors [5, 28, 31, 52, 58, 76, 80, 81, 82, 86].

### Chemical analysis

**FIGURE 7:**
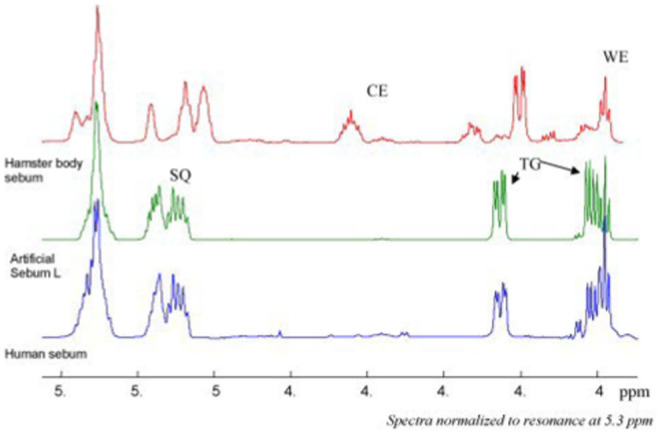
NMR of human, artificial and hamster body sebum. Reprinted from Lu et al. [15]. Copyright (2023) Elsevier.

Nuclear magnetic resonance (NMR) has been used to characterize six formulations in three publications [15, 47, 66], although the NMR results were not published in two of them. However, the authors stated that artificial sebum exhibited good correlation to human sebum [47, 66]. Lu et al. [15] compared the NMR data among human sebum, hamster sebum and artificial sebum prepared by them, and identified peaks at 5.04–5.13 ppm for squalene (SQ), 4.52–4.60 ppm for cholesterol esters (CE), 4.20–4.30 ppm for triglycerides (TR) and 3.96–3.99 for wax esters (WE) (Figure 7) [15]. It is interesting to note that hamster sebum contained a peak for cholesterol esters, while human and artificial sebum did not show such a peak. The composition of cholesterol esters in human sebum is usually very small (2.0 ± 0.5 wt%) [106]. The peaks for squalene, wax esters and triglycerides were in the same regions for human and artificial sebum, which led the authors to conclude that their artificial sebum formulation was an appropriate alternative for human sebum [15]. Fatty acids and cholesterol, the remaining two important components of sebum, were not identified in the NMR spectra. These results are consistent with the NMR spectra of human and hamster sebum obtained by Robosky et al. [16]. Fatty acids and cholesterol are complex lipids known to be very challenging in terms of NMR analysis due to their overlapping signal peaks [107]. It was not until 2017 when Alexandri et al. used high‐resolution NMR spectroscopic techniques in analysis of unsaturated lipids that were able to characterize fatty acids and esters that are commonly found in nature [107].

### Stability

**FIGURE 8:**
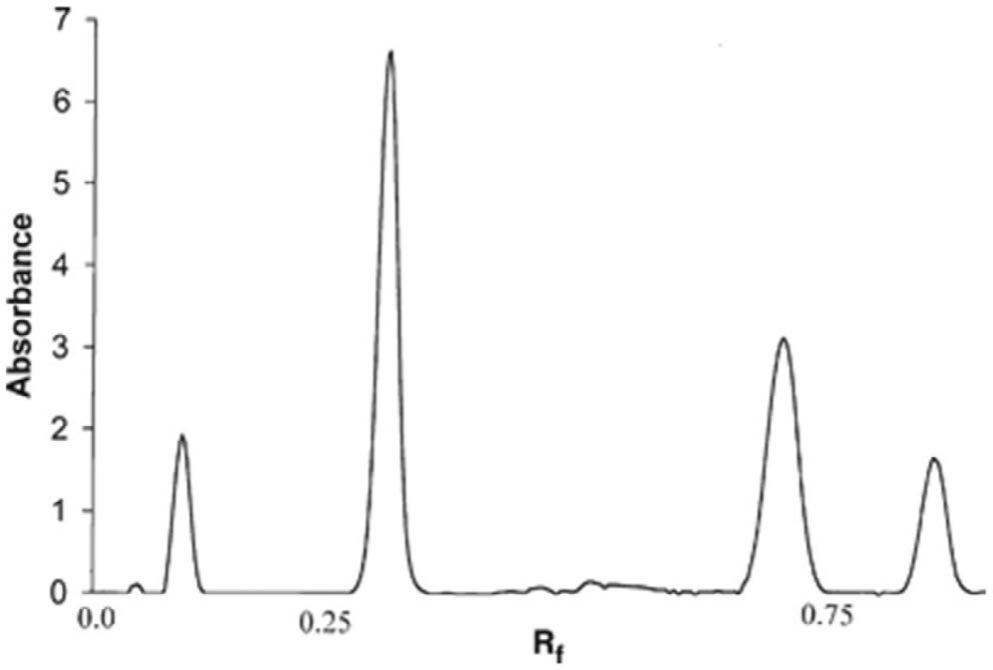
Carbon density profile of a TLC of artificial sebum. Reprinted from Wertz [30]. Copyright (2024) John Wiley and Sons.

Thin‐layer chromatography (TLC) has been used to evaluate sebum's stability by determining the composition of sebum when exposed to ageing. However, only two of 81 formulations have been characterized using this technique [30, 49]. Similar to the authors who used TLC for characterization of artificial sebum. Wertz [30] prepared a standardized formulation of artificial sebum and tested it for stability using TLC. The typical carbon density profile of artificial sebum contains four peaks, which are at Rf values of around 0.10 (fatty acid), 0.30 (triglyceride), 0.70 (wax monoester) and 0.85 (squalene). The area underneath the curves corresponds to the weight percentage of each component in the artificial sebum, as shown in Figure 8 [30]. Based on the differences in the area under the curves, the stability of each component could be determined. In the suggested formulation, cholesterol or cholesterol esters were not included but vitamin E was [30]. The authors suggested that the measurements are the same with or without vitamin E. This is due to the presence of wax monoester (jojoba oil) that is rich in anti‐oxidants and therefore addition of vitamin E in artificial sebum is unnecessary [30]. However, Stefaniak et al. (2010) highlight that when sebum is stored at 32°C squalene oxidizes rapidly in the absence of vitamin E [49].

### Tribology

The coefficient of friction (CoF) is a dimensionless number often used in tribology (the study of friction, wear and lubrication) to quantify the friction between two surfaces. It is calculated by using the ratio of frictional force to the normal force applied on the surface.

The CoF of artificial sebum was measured in only three studies. In all three papers, sebum was first placed on a substrate such as hair swatches or a skin model prior to the measurement. Bhuyan et al. [56] measured the CoF of fatty acids (oleic and stearic acid) that are often used in artificial sebum. However, the acids were not part of a complex sebum mixture and were measured on steel to evaluate their lubrication performance. Stearic and oleic acid reduce the CoF by 38.3% and 34.2%, respectively, with respect to the base oil [56]. They found that acids reduce CoF better than the esters tested, for example, methyl oleate [56].

Gerhardt et al. (2009) studied lipid‐coated skin models that simulate skin lubrication by artificial sebum on a skin model. It was characterized in terms of physiochemical and tribological properties [48]. Concentrations studied ranged between 0 (control) and 100 μg cm^−2^ of artificial sebum on the skin model. The CoF of the control skin model was 0.61 and application of artificial sebum resulted in a CoF of 0.43 with 25 μg cm^−2^. The final CoF varied between 0.62 for model skin with 100 μg cm^−2^ lipid mixture and 0.94 for the control skin model, depending on the number of frictional cycles [48]. In general, the higher the friction forces applied, the higher the CoF and the system consisting of model skin and artificial sebum lay in the boundary lubrication regime of the Stribeck curve [48]. As artificial sebum is an oily mixture, it therefore acts as a viscous lubricant, which could imply that the surface is better lubricated with a greater amount of sebum on the surface [48]. The authors also examined the effect of sliding velocity and the normal load [48]. Also in 2012, Derler and Gerhardt published a review article on skin tribology, which studied friction on human skin [17].

Korbeld et al. (2020) investigated the effect of sebum composition on the frictional response of skin [28]. They tested five artificial sebum formulations that contained squalene, wax, triglycerides, fatty acids and cholesterol based on sebum formulations reported previously [28]. Cholesterol esters were omitted as they take a small proportion of human sebum [28]. It was found that the composition of sebum significantly affected the friction response, although no mechanism was proposed. The average CoF value for sebum was 0.20 for all formulations tested, while one formulation with a high amount of squalene (20 wt%) gave a high CoF up to 0.80 [28]. The presence of fatty acid at 25 wt% also increased the CoF to 0.27, a smaller change than that for squalene [28]. The variation in composition of sebum affects CoF, where high squalene and high fatty acid content yield very different results to lower concentrations used [28].

Due to the complex composition of the artificial sebum, there is limited understanding of the effect of sebum composition on its tribological characteristics. It is worth noting that individuals who produce sebum with a high concentration of squalene and fatty acids could experience enhanced friction, leading to an increased level of irritation and itchiness. More research is required to understand the effect of the composition of sebum on skin friction.

Galliano et al. [44] measured the CoF on hair swatches covered by a sebum‐like emulsion that consisted of oleic acid (20% v/v) and water (80% v/v), which was exposed to air pollutants for 24 and 72 h. The swatches were covered with more pollutants after 72 h, and the dry sliding force increased by 90% and 100% after 24 and 72 h, respectively (from 0.4 N dry sliding force to 0.7 and 0.8 N) [44]. The results show that there is more frictional resistance when more air pollutants are attached to sebum‐covered hair swatches. In that study, the artificial sebum contained 80% water, which resembled a sebum–sweat environment. Considering how many artificial sebum formulations are available in the literature, experiments could be improved by using more sebum‐like formulations on hair swatches, which would yield more representative results. Nevertheless, this study shows the effect of air pollutants on hair friction.

### Other characterizations

There are five publications in which artificial sebum was characterized using methods other than those described above. Wertz [30] measured the stability of artificial sebum over 48 h at 22°C and 32°C and found that sebum takes up a small amount of water during that time.

**FIGURE 9:**
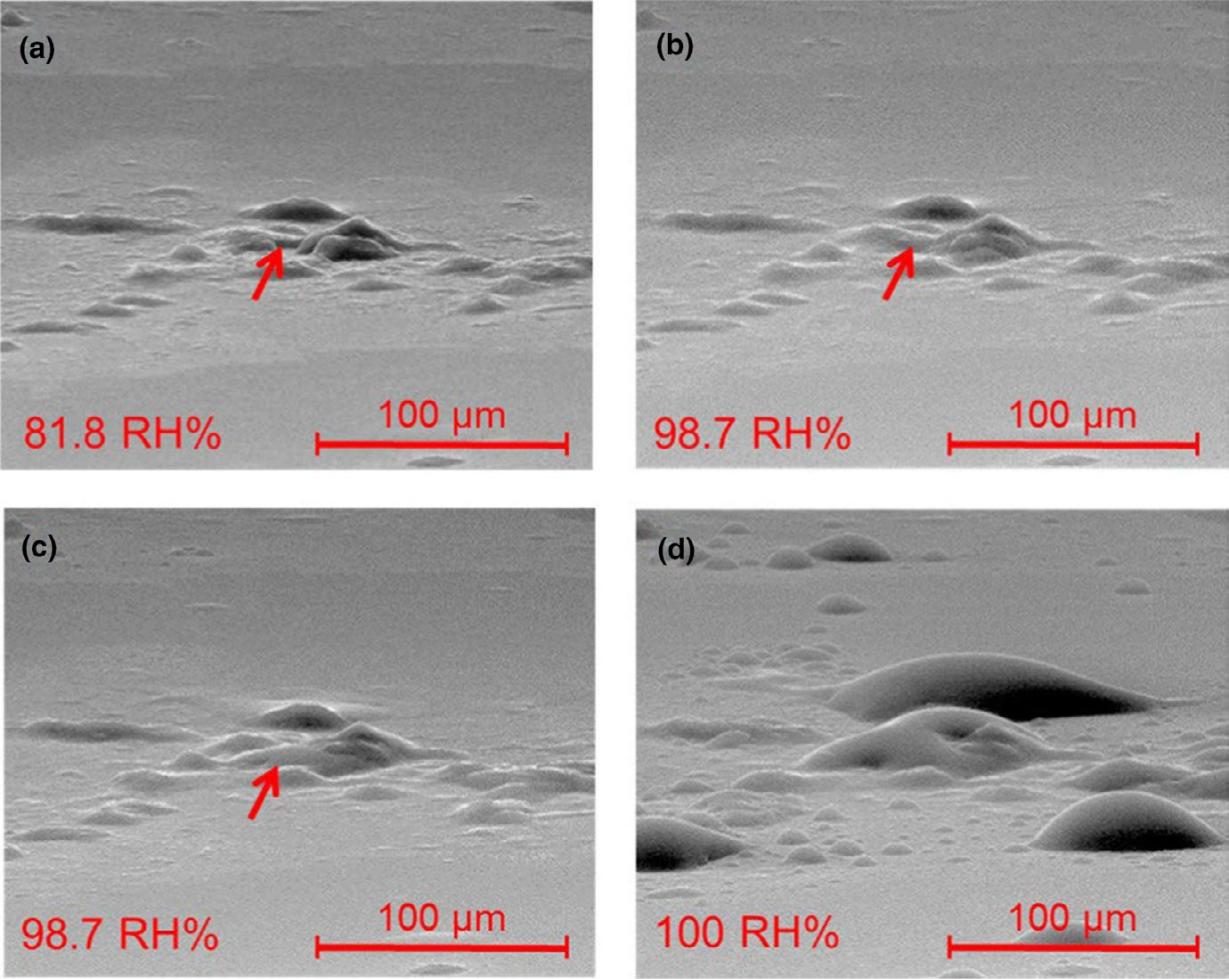
SEM images of sebum swelling due to increasing relative humidity at (a) 81.8% and (b) 98.7% for 7 min to (c) 98.7% for additional 6.5 min and reaching saturation at 100% (d). Reprinted from Stoehr et al. [101] Copyright (2023) American Chemical Society.

Stoehr et al. (2016) used an artificial sebum formulation to form fingerprints on easy‐to‐clean coatings and focused on understanding contamination of the fingerprints due to environmental user‐induced effects [101]. It was found using scanning electron microscopy (SEM) (Figure 9) that natural sebum fingerprints became swollen under increased relative humidity. This was consistent with the results reported by Wertz [30] that sebum takes up a small amount of water. However, the authors speculated that the uptake of water is due to salts from the sweat, which was confirmed using energy‐dispersive X‐ray spectroscopy (EDX) [30, 101]. In addition, rheological measurements of artificial sebum were carried out to examine the behaviour of sebum under applied shear stress. It was found that the artificial sebum was shear thinning, and an amplitude sweep test at low shear rates showed G′ larger than G″, implying gel‐like behaviour [101]. Moreover, it was found that the viscosity of artificial sebum decreases with increasing temperature: the yield point at 25°C is 1300 Pa which drops to 150 Pa at 35°C and sebum is not thixotropic [101].

In 2012, Sakamoto et al. measured the absorption spectra from 200 to 3,000 nm of human and artificial sebum to find the wavelengths that would target sebaceous glands for selective photothermolysis [19]. Natural sebum had weak absorption peaks at 1212 and 1364 nm but strong absorption peaks at 1720 and 1760, 2306 and 2346 nm, whereas the peaks for artificial sebum were present at 1210, 1390, 1414, 1728, 1760, 2306 and 2346 nm [19].

One area of interest in recent years is developing an artificial skin model to mimic the surface properties of human skin [108, 109, 110]. For example, to get the most accurate skin mimic, sebum must be applied to it. This was done by Eudier et al. [110] in preparing a nonbiological skin model (NBSM). The artificial sebum was applied to the skin mimic using two protocols, one by spraying sebum solution in a solvent (CHCl_3_:MeOH) and direct deposition at 70°C [110]. Both sebum films were characterized using optical and polarized microscopy, which showed that the one prepared using the first protocol led to the crystallization of the lipids. This was felt to possibly result from solvent evaporation as no crystals were observed with the thin film prepared using the second protocol [110]. Surface free energy was calculated for artificial sebum as well as NBSM thin films using both protocols; artificial sebum had the highest surface free energy (*γ*) of around 45 mJ m^−2^, whereas sebum applied on NBSM showed a reduced *γ* value to 25 and 28 mJ m^−2^ for protocol 1 and protocol 2 respectively [110]. Artificial sebum also has a large Lifschitz‐van der Waals (LW) component value of 41.1 mJ m^−2^, likely due to the interaction between triglycerides and other lipids, making surface cohesion greater and increasing surface energy by apolar interactions [110]. On top of that, infrared (IR) measurements conducted showed peaks for artificial sebum at 2914.65 (CH_2_ asymmetric stretch), 2849.06 (CH_2_ symmetric stretch), 1735.33 (C–O esters) and 1710.27 (C–O fatty acids) cm^−1^.

The surface tension of an artificial sebum formulation was determined by Mo et al. (2007) to be around 58 dyn/cm, whereas human sebum surface tension is 25 dyn/cm at equilibrium [18]. Gerhardt et al. (2009) measured the contact angle of artificial sebum on a skin model and reported a gradual increase in contact angle with increasing lipid concentration [48]. The increased hydrophobicity observed might be due to a random organization of the upper lipid layer as a result of the spontaneous rearrangement of lipids upon exposure to air [48, 111].

**FIGURE 10:**
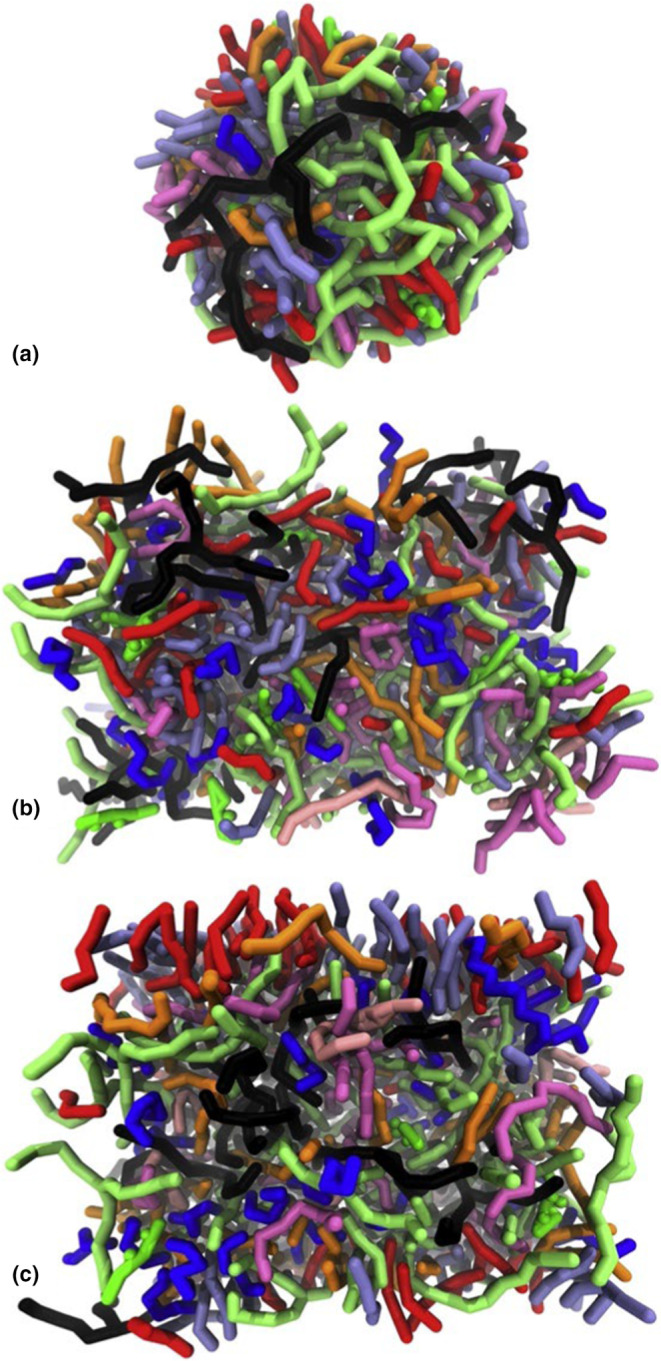
Molecular dynamics model of sebum in water (a), sebum in vacuum (b) and sebum in vacuum after simulations in water (c). Corresponding colours are squalene: blue, palmitic acid: red, palmitoleic acid: ice‐blue, palmityl palmitate: orange, oleyl oleate: mauve, tripalmitin: lime, triolein: black, cholesterol: green and cholesterol oleate: pink. Reprinted from Antunes et al. [58]. Copyright (2023) Elsevier.

Most knowledge about artificial sebum comes from studies examining its physical, chemical and mechanical properties. However, Antunes and Cavaco‐Paulo (2020) took a different approach to molecular dynamics and simulations [58]. This was the first molecular dynamics (MD) model of sebum. It was reported that sebum in water assembled into a spherical structure, but it created an unstructured layer when placed in vacuum (Figure 10) [58]. When in water, the free fatty acids are present more on the surface with the acid groups facing water and acyl chains hidden in the spherical structure [58]. This MD model was subsequently used to examine the interactions of sebum with other compounds such as Nile red, polylactic acid and poloxamer polymers [58]. Establishing a molecular model to simulate sebum could be useful for penetration and dermatological studies.

All the characterization data for the artificial sebum must be treated with caution, as each measurement was conducted using a slightly different formulation. As shown before in CoF part, the addition of various concentrations of squalene or fatty acids changes the results substantially. There is no standard artificial sebum formulation that is used in research and the physiochemical properties of each sebum therefore differ.

## COMPARISON WITH HUMAN SEBUM

Artificial sebum should resemble human sebum as closely as possible. The comparison of the properties of artificial sebum and human sebum is presented in Table 6. In general, the physiochemical properties of human and artificial sebum are similar. However, various authors have measured the properties of different artificial sebum formulations, and the human sebum tested is always taken from different individuals.

**TABLE 6:** Comparison of physiochemical characteristics of human and artificial sebum.

Measurement	Artificial sebum	Human sebum
DSC	Melting points at −20°C, 15°C, 40°C and 55°C [55] Melting points at 30.63°C and 36.07°C [47, 66] Melting points at 33.3°C and 40.28°C [15]	Sharp peaks at −25°C and 25°C [14] Melting points at 32.9°C and 42.0°C [15]
NMR	Peaks at: 5.04–5.13 ppm squalene, 4.20–4.30 ppm triglycerides and 3.96–3.99 wax esters [15]	Peaks at: 5.04–5.13 ppm squalene, 4.20–4.30 ppm triglycerides and 3.96–3.99 wax esters [15] Peaks at: 5.06–5.13 ppm squalene, 4.52–4.60 ppm cholesterol esters, 4.21–4.30 ppm triglycerides and 3.96–3.99 wax esters [16]
TLC	Rf peaks at 0.10 fatty acids, 0.30 triglycerides, 0.70 wax monoester and 0.85 squalene [30]	TLC of sebum with the order of sebum components: free fatty acids (bottom), triglycerides (middle), wax esters, cholesterol esters and squalene (top). No Rf values were given, however, extract was analysed using GC‐FID [4]
CoF	CoF varies between 0.62 and 0.94 with varying amount of sebum applied. The more sebum on the skin, the better the lubrication [48]. Squalene increases CoF, composition of sebum has a great effect on the CoF [28]	No information on CoF of sebum itself on skin, however, many publications on skin tribology, refer to Derler and Gerhardt (2012) [17]
Surface tension	58 dyn/cm at room temperature [18]	25 dyn/cm at 30°C and room temperature [12, 18]
IR	2914.65 cm^−1^ (CH_2_ asymmetric stretch), 2849.06 (CH_2_ symmetric stretch), 1735.33 (C–O esters) and 1710.27 (C–O fatty acids) cm^−1^ [110]	1740 cm^−1^ (C–O fatty esters), 1710 cm^−1^ (C–O fatty acids) and 1460 cm^−1^ (CH_2_ hydrocarbons) [10]
Absorption	Absorption peaks at: 1210, 1390, 1414, 1728, 1760, 2306 and 2326 nm [19]	Absorption peaks at: 1212, 1364 nm (weak), 1720, 1760, 2306, 2346 nm (strong) [19]
Rheology	Shear thinning behaviour at low shear rates G′ < G″. Viscosity decreases with increasing temperature. Yield point at 25°C is 1300 Pa and at 35°C 150 Pa. No thixotropic behaviour [101]	Viscosity 997.5 millipoises at 26.5°C and 551.9 millipoises at 38°C [12]

The reported melting points of human sebum are different from those measured on artificial sebum. This is probably expected because preparing artificial sebum with the same composition as human sebum will be almost impossible due to variability. It was recommended that melting temperature offers a good indication of how closely artificial sebum is to human sebum.

NMR is a good technique for distinguishing specific components of sebum, and the reported NMR shifts for squalene, wax esters and triglycerides of artificial sebum lie in the same region as human sebum.

TLC analysis provides an indication on the general class of chemical components present in sebum, and the TLC bands were found similar for artificial and human sebum. However, they only provide an indication on what group of components is present in the sebum.

The notable differences between artificial and human sebum are in terms of surface tension [12, 18], CoF [48] and rheological information [12, 101]. The reported values of surface tension differ between human and artificial sebum, although only two publications [12, 18] report those values at different temperatures, which explains such a big difference in surface tension. There is no research into the effect of the composition of sebum on its surface tension. The surface tension will, however, be crucial when understanding the interactions of sebum with surfaces such as fabrics.

Human sebum has not been tested for just CoF; however, there have been many experiments focusing on skin tribology. These lack knowledge of tribological properties of sebum and how sebum composition affects friction. In terms of rheological properties, very little research has been carried out to understand the behaviour of sebum. The similarity is that both artificial and human sebum were reported to have decreasing viscosity with increasing temperature.

The IR stretching bands of human and artificial sebum are similar for both human and artificial sebum, however, the reported values for human sebum only cover short range of wavenumbers. Considering sebum is an organic material, there are no differences to be expected in the IR spectra.

## CONCLUSIONS

This review has surveyed the artificial sebum formulations used in various research areas and the corresponding physicochemical properties. Artificial sebum has been prepared with a wide range of compounds that are representative of the primary components identified in natural sebum. Although various formulations were used, only limited physicochemical characterization has been carried out, which led to variability in results. It is challenging to define a standardized artificial sebum formulation as human sebum differs between individuals.

The currently used artificial sebum is poorly characterized and is often not representative of human sebum. From the formulations that have been characterized, the physicochemical properties are not well understood and more research is needed. The fundamental knowledge of surface interactions lies in understanding the material that gets deposited on the surfaces, which is why sebum, as a complex material, needs to be investigated in much more detail. The interactions of sebum with surfaces such as fabrics, skin, hair, electronics or high‐traffic surfaces would lead to a better development of home, personal care products and anti‐fingerprint coatings. It is unlikely that a standardized formulation for artificial sebum will be agreed or developed, largely because of the considerable variations observed between individual human beings. However, it might be possible to utilize artificial intelligence and chemical information acquired from different demographic groups to establish a suite of consistent formulations of artificial sebum.

Based on the current understanding of artificial sebum, several avenues for further research can be proposed. An increasing understanding of drug and small molecule penetration through sebum into the skin facilitates the development of more effective and personalized pharmaceuticals. Additionally, insights into bacterial growth on sebum can inform the formulation of tailored skincare solutions. The composition of sebum significantly influences the efficacy of cosmetic products applied to the skin. Thus, the expanding knowledge of the interactions among sebum, molecules and microorganisms holds substantial potential for advancing personalized drug and skincare development.

Research has shown that sebum absorbs preferentially to hydrophobic materials, such as polyester, and that high concentrations of squalene result in stains that are more challenging to remove. This understanding can inform the development of home care products specifically designed to target and clean hydrophobic materials more effectively. Additionally, increased awareness of sebum‐related staining and odour formation may influence consumer purchasing behaviour.

Lastly, we would like to highlight that particular attention should be made towards the composition of artificial sebum, and consequently its interactions with the surrounding environment when performing studies using artificial sebum.

## CONFLICT OF INTEREST STATEMENT

The authors report no conflict of interest.

## Supporting information


Table S1.

## References

[1] Grice EA , Segre JA . The skin microbiome. Nat Rev Microbiol. 2011;9:244–253. 21407241 10.1038/nrmicro2537 PMC3535073

[2] Kong HH , Segre JA . Skin microbiome: looking Back to move forward. J Invest Dermatol. 2012;132:933–939. 22189793 10.1038/jid.2011.417 PMC3279608

[3] Biggs LC , Kim CS , Miroshnikova YA , Wickström SA . Mechanical forces in the skin: roles in tissue architecture, stability, and function. J Invest Dermatol. 2020;140:284–290. 31326398 10.1016/j.jid.2019.06.137

[4] Pappas A , Johnsen S , Liu JC , Eisinger M . Sebum analysis of individuals with and without acne. Derm‐Endocrinol. 2009;1:157–161. 10.4161/derm.1.3.8473 PMC2835908 20436883

[5] Champmartin C , Chedik L , Marquet F , Cosnier F . Occupational exposure assessment with solid substances: choosing a vehicle for in vitro percutaneous absorption experiments. Crit Rev Toxicol. 2022;52:294–316. 36125048 10.1080/10408444.2022.2097052

[6] Landemaine L , Da Costa G , Fissier E , Francis C , Morand S , Verbeke J , et al. Staphylococcus epidermidis isolates from atopic or healthy skin have opposite effect on skin cells: potential implication of the AHR pathway modulation. Front Immunol. 2023;14:1098160. 37304256 10.3389/fimmu.2023.1098160 PMC10250813

[7] Nordstrom KM , Labows JN , McGinley KJ , Leyden JJ . Characterization of wax esters, triglycerides, and free fatty acids of follicular casts. J Invest Dermatol. 1986;86:700–705. 2940302 10.1111/1523-1747.ep12276314

[8] Picardo M , Ottaviani M , Camera E , Mastrofrancesco A . Sebaceous gland lipids. Derm‐Endocrinol. 2009;1:68–71. 10.4161/derm.1.2.8472 PMC2835893 20224686

[9] Downing DT , Strauss JS , Pochi PE . Variability in the chemical composition of human skin surface lipids. J Invest Dermatol. 1969;53:322–327. 5347411 10.1038/jid.1969.157

[10] Anderson AS , Fulton JE . Sebum: analysis by infrared spectroscopy. J Invest Dermatol. 1973;60:115–120. 4266981 10.1111/1523-1747.ep12682018

[11] Steiner R , Roux C , Moret S . Controlling fingermark variability for research purposes: a review. WIREs Forensic Sci. 2019;1:e1338.

[12] Butcher EO , Coonin A . The physical properties of human sebum. J Invest Dermatol. 1949;12:249–254. 18120703

[13] Greene RS , Downing DT , Pochi PE , Strauss JS . Anatomical variation in the amount and composition of human skin surface lipid. J Invest Dermatol. 1970;54:240–247. 5436951 10.1111/1523-1747.ep12280318

[14] Bore P , Goetz N , Caron JC . Differential thermal analysis of human sebum as a new approach to rheological behaviour. Int J Cosmet Sci. 1980;2:177–191. 19467091 10.1111/j.1467-2494.1980.tb00244.x

[15] Lu GW , Valiveti S , Spence J , Zhuang C , Robosky L , Wade K , et al. Comparison of artificial sebum with human and hamster sebum samples. Int J Pharm. 2009;367:37–43. 18929636 10.1016/j.ijpharm.2008.09.025

[16] Robosky LC , Wade K , Woolson D , Baker JD , Manning ML , Gage DA , et al. Quantitative evaluation of sebum lipid components with nuclear magnetic resonance. J Lipid Res. 2008;49:686–692. 18094397 10.1194/jlr.D700035-JLR200

[17] Derler S , Gerhardt LC . Tribology of skin: review and analysis of experimental results for the friction coefficient of human skin. Tribol Lett. 2012;45:1–27.

[18] Mo YK , Kankavi O , Masci PP , Mellick GD , Whitehouse MW , Boyle GM , et al. Surfactant protein expression in human skin: evidence and implications. J Invest Dermatol. 2007;127:381–386. 17008883 10.1038/sj.jid.5700561

[19] Sakamoto FH , Doukas AG , Farinelli WA , Tannous Z , Shinn M , Benson S , et al. Selective photothermolysis to target sebaceous glands: theoretical estimation of parameters and preliminary results using a free electron laser. Lasers Surg Med. 2012;44:175–183. 22170298 10.1002/lsm.21132

[20] Honari G , Maibach H . Chapter 1 – skin structure and function. In: Maibach H , Boston HG , editors. Applied Dermatotoxicology. Cambridge, MA, USA: Academic Press; 2014. p. 1–10.

[21] Colin‐Russ A . Human foot perspiration, its nature and interactions with footwear. J Hyg. 1935;35:199–206. 20475272 10.1017/s0022172400032216 PMC2170765

[22] Colin‐Russ A . Human foot perspiration and upper leather. Epidemiol Infect. 1940;40:447–452. 10.1017/s0022172400027947 PMC2199705 20475555

[23] Colin‐Russ A . Further studies on foot perspiration and its action on footwear. J Hyg. 1943;43:72–82. 20475662 10.1017/s0022172400035877 PMC2234657

[24] Colin‐Russ A . On a new form of reagent against perspiration effects on shoe materials. J Hyg. 1945;44:53–55. 20475712 10.1017/s0022172400013292 PMC2234723

[25] Roddy WT , Lollar RM . Resistance of white leather to breakdown by perspiration. J Am Leather Chem Assoc. 1955;50:180–192.

[26] Spangler WG , Cross HD , Schaafsma BR . A laboratory method for testing laundry products for detergency. J Am Oil Chem Soc. 1965;42:723–727.

[27] Stefaniak AB , Harvey CJ . Dissolution of materials in artificial skin surface film liquids. Toxicol In Vitro. 2006;20:1265–1283. 16860531 10.1016/j.tiv.2006.05.011

[28] Korbeld KT , Klaassen M , Jobanputra RD , Vries EG , Masen MA . Effects of sebum properties on skin friction: investigation using a bench test. Biosurf Biotribol. 2020;6:43–47.

[29] Blanc D , Saint‐Leger D , Brandt J , Constans S , Agache P . An original procedure for quantitation of cutaneous resporption of sebum. Arch Dermatol Res. 1989;281:346–350. 2802665 10.1007/BF00412980

[30] Wertz PW . Human synthetic sebum formulation and stability under conditions of use and storage. Int J Cosmet Sci. 2009;31:21–25. 19134124 10.1111/j.1468-2494.2008.00468.x

[31] Parker K , Morrison G . Methamphetamine absorption by skin lipids: accumulated mass, partition coefficients, and the influence of fatty acids. Indoor Air. 2016;26:634–641. 26126994 10.1111/ina.12229

[32] Druart ME , Recloux I , Derouaux A , Van De Van De Weerdt C , Olivier MG . Development of mesoporous sol–gel films for antifingerprint applications on glass. J Sol‐Gel Sci Technol. 2018;88:334–344.

[33] Marin Villegas CA , Guney M , Zagury GJ . Comparison of five artificial skin surface film liquids for assessing dermal bioaccessibility of metals in certified reference soils. Sci Total Environ. 2019;692:595–601. 31539967 10.1016/j.scitotenv.2019.07.281

[34] De La Hunty MA . An investigation of latent fingermark residues and their de‐ velopment on porous substrates using physical developer and nile red. Thesis. 2017.

[35] Yokoi A , Endo K , Ozawa T , Miyaki M , Matsuo K , Nozawa K , et al. A cleanser based on sodium laureth carboxylate and alkyl carboxylates washes facial sebum well but does not induce dry skin. J Cosmet Dermatol. 2014;13:245–252. 25399616 10.1111/jocd.12118

[36] Peterson G , Rapaka S , Koski N , Kearney M , Ortblad K , Tadlock L . A robust sebum, oil, and particulate pollution model for assessing cleansing efficacy of human skin. Int J Cosmet Sci. 2017;39:351–354. 27797421 10.1111/ics.12378

[37] Galliano A , Ye C , Su F , Wang C , Rakshit R , Guerin M , et al. Assessing the effect of cleansing products on artificially polluted human hairs and skin through in vivo and in vitro models. Skin Res Technol. 2023;29:e13220. 36609868 10.1111/srt.13220 PMC10155848

[38] Nelson C , Braaten A , Fleekert J . The effect of synthetic dermal secretion on transfer and dissipation of the insecticide aldicarb from granular formulation to fabric. Arch Environ Contam Toxicol. 1993;24:513–516. 8507108 10.1007/BF01146171

[39] Skopp G , Pötsch L , Moeller MR . On cosmetically treated hair — aspects and pitfalls of interpretation. Forensic Sci Int. 1997;84:43–52. 9042709 10.1016/s0379-0738(96)02047-6

[40] Mainkar AR , Jolly CI . Evaluation of commercial herbal shampoos. Int J Cosmet Sci. 2000;22:385–391. 18503425 10.1046/j.1467-2494.2000.00047.x

[41] Musial W , Kubis A . Preliminary assessment of alginic acid as a factor buffering triethanolamine interacting with artificial skin sebum. Eur J Pharm Biopharm. 2003;55:237–240. 12637103 10.1016/s0939-6411(02)00195-9

[42] Katsuta Y , Iida T , Inomata S , Denda M . Unsaturated fatty acids induce calcium influx into keratinocytes and cause abnormal differentiation of epidermis. J Invest Dermatol. 2005;124:1008–1013. 15854043 10.1111/j.0022-202X.2005.23682.x

[43] Sisco E , Staymates J , Schilling K . A chemically relevant artificial fingerprint material for the cross‐comparison of mass spectrometry techniques. J Can Soc Forensic Sci. 2015;48:200–214.

[44] Galliano A , Ye C , Su F , Wang C , Wang Y , Liu C , et al. Particulate matter adheres to human hair exposed to severe aerial pollution: consequences for certain hair surface properties. Int J Cosmet Sci. 2017;39:610–616. 28748540 10.1111/ics.12416

[45] Spittaels KJ , Coenye T . Developing an in vitro artificial sebum model to study Propionibacterium acnes biofilms. Anaerobe. 2018;49:21–29. 29175428 10.1016/j.anaerobe.2017.11.002

[46] Suzuki K , Inoue M , Takayama K , Nagahama T , Cho O , Kurakado S , et al. Development of artificial‐sebum‐containing Leeming and Notman agar medium to enhance the growth of Malassezia. Mycopathologia. 2022;187:393–396. 35610393 10.1007/s11046-022-00634-9

[47] Valiveti S , Lu GW . Diffusion properties of model compounds in artificial sebum. Int J Pharm. 2007;345:88–94. 17624701 10.1016/j.ijpharm.2007.05.043

[48] Gerhardt LC , Schiller A , Müller B , Spencer ND , Derler S . Fabrication, Char‐acterisation and Tribological investigation of artificial skin surface lipid films. Tribol Lett. 2009;34:81–93.

[49] Stefaniak AB , Harvey CJ , Wertz PW . Formulation and stability of a novel artificial sebum under conditions of storage and use. Int J Cosmet Sci. 2010;32:347–355. 20491993 10.1111/j.1468-2494.2010.00561.x

[50] Pawar G , Abdallah MAE , de Sáa EV , Harrad S . Dermal bioaccessibility of flame retardants from indoor dust and the influence of topically applied cosmetics. J Expo Sci Environ Epidemiol. 2017;27:100–105. 26732374 10.1038/jes.2015.84

[51] Luo K , Zeng D , Kang Y , Lin X , Sun N , Li C , et al. Dermal bioaccessibility and absorption of polycyclic aromatic hydrocarbons (PAHs) in indoor dust and its implication in risk assessment. Environ Pollut. 2020;264:114829. 32559865 10.1016/j.envpol.2020.114829

[52] Pannu J , McCarthy A , Martin A , Hamouda T , Ciotti S , Ma L , et al. In vitro antibacterial activity of NB‐003 against Propionibacterium acnes. Antimicrob Agents Chemother. 2011;55:4211–4217. 21746943 10.1128/AAC.00561-11 PMC3165361

[53] Kostrzebska A , Paczek K , Weselak A , Musial W . Effect of hydrogel substrate components on the stability of tetracycline hydrochloride and swelling activity against model skin sebum. Int J Mol Sci. 2023;24:2678. 36768998 10.3390/ijms24032678 PMC9916833

[54] Arsenault H , Nic Daeid N , Gray A . A synthetic fingerprint solution and its importance in DNA transfer, persistence and recovery studies. Forensic Sci Int: Synergy. 2023;6:100330. 37249970 10.1016/j.fsisyn.2023.100330 PMC10209804

[55] Motwani MR , Rhein LD , Zatz JL . Differential scanning calorimetry studies of sebum models. J Cosmet Sci. 2001;52(4):211–224. 11479654

[56] Bhuyan S , Sundararajan S , Yao L , Hammond EG , Wang T . Boundary lubrication properties of lipid‐based compounds evaluated using microtribological methods. Tribol Lett. 2006;22:167–172.

[57] Kattou P , Lian G , Glavin S , Sorrell I , Chen T . Development of a two‐ dimensional model for predicting transdermal permeation with the follicular pathway: demonstration with a caffeine study. Pharm Res. 2017;34:2036–2048. 28660400 10.1007/s11095-017-2209-0 PMC5579157

[58] Antunes E , Cavaco‐Paulo A . Stratum corneum lipid matrix with unusual packing: a molecular dynamics study. Colloids Surf B Biointerfaces. 2020;190:110928. 32179416 10.1016/j.colsurfb.2020.110928

[59] Schneider D , Dennerlein K , Göen T , Schaller KH , Drexler H , Korinth G . Influence of artificial sebum on the dermal absorption of chemicals in excised human skin: A proof‐of‐concept study. Toxicol In Vitro. 2016;33:23–28. 26911728 10.1016/j.tiv.2016.02.010

[60] Hemingway JD , Molokhia M . The dissolution of metallic nickel in artificial sweat. Contact Derm. 1987;16:99–105. 10.1111/j.1600-0536.1987.tb01388.x 3568645

[61] Alkilani A , McCrudden MT , Donnelly R . Transdermal drug delivery: innovative pharmaceutical developments based on disruption of the barrier properties of the stratum Corneum. Pharmaceutics. 2015;7:438–470. 26506371 10.3390/pharmaceutics7040438 PMC4695828

[62] Stefaniak AB , Virji MA , Day GA . Release of beryllium from beryllium‐ containing materials in artificial skin surface film liquids. Ann Occup Hyg. 2011;55:57–69. 20729394 10.1093/annhyg/meq057

[63] Marin Villegas CA , Zagury GJ . Comparison of synthetic sweat and influence of sebum in the permeation of bioaccessible metal(loid)s from contaminated soils through a synthetic skin membrane. Environ Sci Technol. 2021;55:8215–8222. 34039002 10.1021/acs.est.1c02038

[64] Ghislain FA , Zagury GJ . Influence of sebum proportion in synthetic sweat on dermal bioaccessibility and on permeation of metal(loid)s from contaminated soils. Environ Sci Pollut Res. 2023;30:86762–86772. 10.1007/s11356-023-28388-x 37414993

[65] Motwani MR , Rhein LD , Zatz JL . Influence of vehicles on the phase transitions of model sebum. J Cosmet Sci. 2002;53:35–42. 11917254

[66] Valiveti S , Wesley J , Lu GW . Investigation of drug partition property in artificial sebum. Int J Pharm. 2008;346:10–16. 17651932 10.1016/j.ijpharm.2007.06.001

[67] Yang S , Li L , Chen T , Han L , Lian G . Determining the effect of pH on the partitioning of neutral, cationic and anionic chemicals to artificial sebum: new physicochemical insight and QSPR model. Pharm Res. 2018;35:141. 29761237 10.1007/s11095-018-2411-8

[68] Yang S , Li L , Lu M , Chen T , Han L , Lian G . Determination of solute diffusion properties in artificial sebum. J Pharm Sci. 2019;108:3003–3010. 31054887 10.1016/j.xphs.2019.04.027

[69] Doran GS , Howitt JA . Bioaccessibility of drug residues on common Police Station work surfaces. J Anal Toxicol. 2018;43:144–148. 10.1093/jat/bky073 30295840

[70] Musial W , Kubis A . Carbopols as factors buffering triethanoloamine interacting with artificial skin sebum. Polim Med. 2004;34:17–30. 15850295

[71] Musial W , Kubis A . Preliminary evaluation of interactions between selected Alcoholamines and model skin sebum components. Chem Pharm Bull. 2006;54:1076–1081. 10.1248/cpb.54.1076 16880647

[72] Hatamleh MM , Watts DC . Effect of Extraoral aging conditions on color stability of maxillofacial silicone elastomer. J Prosthodont. 2010;19:536–543. 20723020 10.1111/j.1532-849X.2010.00627.x

[73] Hatamleh MM , Polyzois GL , Silikas N , Watts DC . Effect of Extraoral Aging conditions on mechanical properties of maxillofacial silicone elastomer. J Prosthodont. 2011;20:439–446. 21777333 10.1111/j.1532-849X.2011.00736.x

[74] Hatamleh MM , Watts DC . Porosity and color of maxillofacial silicone Elastomer. J Prosthodont. 2011;20:60–66. 21054642 10.1111/j.1532-849X.2010.00652.x

[75] Reifenrath W , Ross J , Maas W , Conti J , Driver JH , Bartels M . Estimated dermal penetration of Tetrachlorvinphos (TCVP) in humans based on in Silico modeling and in vitro and in vivo data. J Toxicol Env Heal A. 2023;86:1–13. 10.1080/15287394.2023.2208593 37203870

[76] Abdel‐Mottaleb MMA , Beduneau A , Pellequer Y , Lamprecht A . Stability of fluorescent labels in PLGA polymeric nanoparticles: quantum dots versus organic dyes. Int J Pharm. 2015;494:471–478. 26307264 10.1016/j.ijpharm.2015.08.050

[77] Kim DY , Sochichiu S , Kwon JH . Effects of time, temperature, and sebum layer on migration rate of plasticizers in polyvinyl chloride products. Chemosphere. 2022;308:136478. 36122749 10.1016/j.chemosphere.2022.136478

[78] Bajagain R , Panthi G , Park JH , Moon JK , Kwon J , Kim DY , et al. Enhanced migration of plasticizers from polyvinyl chloride consumer products through artificial sebum. Sci Total Environ. 2023;874:162412. 36858231 10.1016/j.scitotenv.2023.162412

[79] Champmartin C , Marquet F , Chedik L , Chedik L , Décret MJ , Aubertin M , et al. Human in vitro percutaneous absorption of bisphenol S and bisphenol a: a comparative study. Chemosphere. 2020;252:126525. 32220717 10.1016/j.chemosphere.2020.126525

[80] Baalbaki NH , Kasting GB . The influence of cellulosic coacervate composition on the flux of an entrained agent through a coacervate/sebum barrier. J Control Release. 2017;266:346–354. 28958853 10.1016/j.jconrel.2017.09.032

[81] Baalbaki NH , Kasting GB . Evaluating the transport kinetics of a model compound released from cellulosic Coacervate compositions into artificial sebum. J Pharm Sci. 2017;106:1578–1585. 28259765 10.1016/j.xphs.2017.02.025

[82] Lauterbach A , Mueller‐Goymann CC . Development, formulation, and charac‐ terization of an adapalene‐loaded solid lipid microparticle dispersion for follicular penetration. Int J Pharm. 2014;466:122–132. 24607219 10.1016/j.ijpharm.2014.02.050

[83] Runnsjö A , Liljedahl S , Sagna D , Ekblad M , Alenfall J . A novel microparticle based formulation for topical delivery of FOL‐005, a small peptide. J Pharm Sci. 2022;111:1309–1317. 35093338 10.1016/j.xphs.2022.01.009

[84] Borrel V , Gannesen AV , Barreau M , Gaviard C , Duclairoir‐Poc C , Hardouin J , et al. Adaptation of acneic and non acneic strains of Cutibacterium acnes to sebum‐like environment. Microbiology. 2019;8:8. 10.1002/mbo3.841 PMC6741132 30950214

[85] Mart'Yanov SV , Gannesen AV , Plakunov VK . A novel simple in vitro system mimicking natural environment for the biofilm cultivation of cutaneous bacteria. Coatings. 2022;12:1923.

[86] Swaney MH , Nelsen A , Sandstrom S , Kalan LR . Sweat and sebum preferences of the human skin microbiota. Microbiol Spectr. 2023;11:e04180‐22. 36602383 10.1128/spectrum.04180-22 PMC9927561

[87] Komesvarakul N , Sanders M , Szekeres E , Acosta E , Faller J , Mentlik T , et al. Microemulsions of triglyceride‐based oils: the effect of co‐oil and salinity on phase diagrams. J Cosmet Sci. 2006;57:309–325. 16957810

[88] Nandy P , Lucas AD , Gonzalez EA , Hitchins VM . Efficacy of commercially available wipes for disinfection of pulse oximeter sensors. Am J Infect Control. 2016;44:304–310. 26589998 10.1016/j.ajic.2015.09.028

[89] Stefaniak AB , Wade EE , Lawrence RB , Arnold ED , Virji MA . Particle transfer and adherence to human skin compared with cotton glove and pre‐moistened polyvinyl alcohol exposure sampling substrates. J Environ Sci Health A. 2021;56:585–598. 10.1080/10934529.2021.1899524 PMC8276042 33720803

[90] Thompson D , Lemaster CB , Allen R , Whittam JH . Evaluation of relative shampoo detergency. J Soc Cosmet Chem. 1985;36:271–286.

[91] Chi YS , Obendorf SK . Effect of fiber substrates on appearance and removal of aged oily soils. J Surfactant Deterg. 2001;4:35–41.

[92] Varanasi A , Obendorf SK , Pedersen LS , Mejldal R . Lipid distribution on textiles in relation to washing with lipases. J Surfactant Deterg. 2001;4:135–146.

[93] Standard Guide for Evaluating Stain Removal Performance in Home Laundering. 1998.

[94] Miracle GS , Randall SL , Liu Z , Brogden DW , Ketcha MM , Good DA , et al. Copper Chelants and antioxidants in laundry detergent formulations reduce formation of malodor molecules on fabrics. J Surfactant Deterg. 2020;23:1125–1134.

[95] Møllebjerg A , Palmén LG , Gori K , Meyer RL . The bacterial life cycle in textiles is governed by fiber hydrophobicity. Microbiol Spectr. 2021;9:e0118521. 34643452 10.1128/Spectrum.01185-21 PMC8515937

[96] Steiner R , Roux C , Moret S . Production of artificial fingermarks. Part I – synthetic secretions formulation. Forensic Sci Int. 2022;331:111166. 34973483 10.1016/j.forsciint.2021.111166

[97] Girod A , Ramotowski R , Weyermann C . Composition of fingermark residue: a qualitative and quantitative review. Forensic Sci Int. 2012;223:10–24. 22727572 10.1016/j.forsciint.2012.05.018

[98] Eisler SFH . Investigation of synthetic fingerprint solutions. Corrosion. 1954;10:237–242.

[99] Staymates JL , Staymates ME , Gillen G . Evaluation of a drop‐on‐demand micro‐dispensing system for development of artificial fingerprints. Anal Methods. 2013;5:180–186.

[100] Johnston A , Rogers K . A study of the intermolecular interactions of lipid components from analogue fingerprint residues. Sci Justice. 2018;58:121–127. 29526263 10.1016/j.scijus.2017.11.004

[101] Stoehr B , McClure S , Höflich A , Al Kobaisi M , Hall C , Murphy PJ , et al. Unusual nature of fingerprints and the implications for easy‐to‐clean coatings. Langmuir. 2016;32:619–625. 26694744 10.1021/acs.langmuir.5b04138

[102] Downing DT , Stewart ME , Wertz PW , Colton SW , Abraham W , Strauss JS . Skin lipids: an update. J Invest Dermatol. 1987;88:2s–6s. 2950180 10.1111/1523-1747.ep12468850

[103] Harvey CJ , LeBouf RF , Stefaniak AB . Formulation and stability of a novel artificial human sweat under conditions of storage and use. In Vitro Toxicol. 2010;24:1790–1796. 10.1016/j.tiv.2010.06.016 20599493

[104] Spangler WG , Roga RC , Cross HD . A detergency test based on rapid aging of unremoved sebum. J Am Oil Chem Soc. 1967;44:728–732.

[105] Friberg SE , Osborne DW . Interaction of a model skin surface lipid with a modified triglyceride. J Am Oil Chem Soc. 1986;63:123–126.

[106] Stewart ME , Downing DT , Pochi PE , Strauss JS . The fatty acids of human sebaceous gland phosphatidylcholine. Biochim Biophys Acta, Lipids Lipid Metab. 1978;529:380–386. 10.1016/0005-2760(78)90082-6 667084

[107] Alexandri E , Ahmed R , Siddiqui H , Choudhary M , Tsiafoulis C , Gerothanassis I . High resolution NMR spectroscopy as a structural and analytical tool for unsaturated lipids in solution. Molecules. 2017;22:1663. 28981459 10.3390/molecules22101663 PMC6151582

[108] Brohem CA , Da Silva Cardeal LB , Tiago M , Soengas MS , De Moraes Barros SB , Maria‐Engler SS . Artificial skin in perspective: concepts and applications. Pigment Cell Melanoma Res. 2011;24:35–50. 21029393 10.1111/j.1755-148X.2010.00786.x PMC3021617

[109] Yun YE , Jung YJ , Choi YJ , Choi JS , Cho YW . Artificial skin models for animal‐free testing. J Pharm Investig. 2018;48:215–223.

[110] Eudier F , Grisel M , Savary G , Picard C . Design of a Lipid‐Coated Polymeric Material Mimic Human Skin Surface Properties: a performing tool to evaluate skin interaction with topical products. Langmuir. 2020;36:4582–4591. 32252530 10.1021/acs.langmuir.0c00133

[111] Albertorio F , Chapa VA , Chen X , Diaz AJ , Cremer PS . The *α*,*α*‐(1→1) linkage of Trehalose is key to Anhydrobiotic preservation. J Am Chem Soc. 2007;129:10567–10574. 17676844 10.1021/ja0731266 PMC2551324

