# Supplementary material for: A review of artificial sebum formulations, their compositions, uses and physicochemical characteristics
Source: Int J Cosmet Sci. 2024 Sep 9;47(1):168–89. doi: 10.1111/ics.13022 (PMC11788007; doi:10.1111/ics.13022)
Supplement: Supplementary file 1 — Table S1. [file ICS-47-168-s001.docx]

Table S1.

| **Index** | **Author (if multiple AS formulations reported in a paper it is indicated by number in brackets)** | **Year** | **Type of formulation** | **Artificial sebum composition (wt% given when reported)** | **Characterisation (what kind)** | **Category** |
| --- | --- | --- | --- | --- | --- | --- |
| **1** | Katsuta *et al.* | 2005 | New | 10% (w/w) in ethanol of: oleic acid, palmitoleic acid, stearic acid, palmitic acid, triolein | No | Penetration/drug adsorption |
| **2** | Motwani *et al.* | 2001 | Based on Downing 1969 | Different ratios of unsaturated:saturated lipds used. Ranges are: Squalene 13%, Wax ester (unsaturated) 0-27%, Wax ester (saturated) 0 - 27%, Triglycerides (unsaturated) 0-43%, Triglycerides (saturated) 0-43%, Fatty acids (unsaturated) 0-17%, Fatty acids (saturated) 0-17% | Yes (DSC) | Study of sebum |
| **3** | Skopp *et al.* | 1997 | New | Additional chemicals added to the sebum and sweat formulations to check the penetration. Artificial sebum composition: squalene 8%, wax estres: wool wax 25%, triglycerides: vaseline 25%, fatty acids: cacao butter 40%, cholesterol 2% | No | Penetration/drug adsorption |
| **4** | Chi and Obendorf | 2001 | Based on American Society of Testing and Materials (ASTM) ASTM D 4265 | Artificial sebum prepared according to American Society of Testing and Materials (ASTM) method D 4265. Sebum composition: squalene, palmitic acid, stearic acid, paraffin oil, olive oil, cholesterol, oleic acid, linoleic acid, coconut oil, spermacetti wax | No | Cleaning/removal |
| **5** | Varanasi *et al.* | 2001 | Based on ASTM D 4265 | Artificial sebum composition (ASTM D 4265) : water, oleic acid (fatty acid), triolein (triglyceride), cholesteryl oleate (cholesterol ester), liquid paraffin (wax ester), squalene, cholesterol | No | Cleaning/removal |
| **6** | Musial and Kubis | 2003 | New | Artificial sebum composition: pork lard 33% (triglycerides), stearic acid 24% (free fatty acids), lanolin 22% (waxes), squalene 12% and cholesterol 4%. 5wt% are missing ? | No | Penetration/drug adsorption |
| **7** | Musial and Kubis | 2006 | no information | Triglyceride mix, stearic acid, lanolin, squalene, cholesterol. No % reported | No | Penetration/drug adsorption |
| **8** | Bhuyan *et al.* | 2006 | no information | Stearic acid and oleic acid measured for friction and viscosity | Yes (CoF) | Study of sebum |
| **9** | Stefaniak and Harvey | 2006 | New | No specific components suggested of the artificial sebum, only levels of each component suggested based on the actual human sebum composition. Squalene 10.6%, wax esters 25%, triglycerides 33%, free fatty acids 28.3%, cholesterol esters 2%, free cholesterol 4%. | No | Penetration/drug adsorption |
| **10** | Mo *et al.* | 2007 | New | Artifiical sebum composition: 15% squalene, 1% cholesterol, 2% cholesterol oleate, 10% palmitoleic acid, 14% tripalmitin, 28% triolein, 25% oleic acid dissolved in hexane:methanol (9:1) | Yes (Surface Tension) | Cosmetic/dermatology |
| **11** | Valiveti and Lu | 2007 | Based on human sebum composition reported in Rosenthal 1964, Greene 1970, Nordstrom 1986 and Walter and Roberts 2002 | Artificial sebum composition: squalene 15%, Paraffin wax 10% (wax ester), spermaceti wax (wax ester) 15%, olive oil (triglycerides) 10, cotton seed oil (triglycerides) 25%, coconut oil (triglycerides) 10%, fatty acids: oleic acid (1.4%), palmitic acid (5%), palmitoleic acid (5%), cholesterol (1.2%), cholesterol oleate (2.4%) | Yes (H NMR and DSC) | Penetration/drug adsorption |
| **12** | Valiveti *et al.* | 2008 | Based on human sebum composition reported in Rosenthal 1964, Greene 1970, Nordstrom 1986 and Wertz 2001 | Artificial sebum composition: squalene 15%, Paraffin wax 10% (wax ester), spermaceti wax (wax ester) 15%, olive oil (triglycerides) 10, cotton seed oil (triglycerides) 25%, coconut oil (triglycerides) 10%, fatty acids: oleic acid (1.4%), palmitic acid (5%), palmitoleic acid (5%), cholesterol (1.2%), cholesterol oleate (2.4%) | Yes (H NMR and DSC) | Penetration/drug adsorption |
| **13** | Lu *et al.* (1) | 2009 | Based on human sebum composition reported in Wertz, 2002; Rosenthal, 1964; Greene et al., 1970; Nordstrom et al., 1986; Stewart et al., 1978 | Artificial sebum composition: squalene 15%, Paraffin wax 10% (wax ester), spermaceti wax (wax ester) 15%, olive oil (triglycerides) 10, cotton seed oil (triglycerides) 25%, coconut oil (triglycerides) 10%, fatty acids: oleic acid (1.4%), palmitic acid (5%), palmitoleic acid (5%), cholesterol (1.2%), cholesterol oleate (2.4%) | Yes (H NMR and DSC) | Penetration/drug adsorption |
| **14** | Lu et al. (2) | 2009 | Based on Nordstrom 1986 | Artificial sebum composiiton: squalene 13%, palmitic acid myristyl ester 27% (wax ester), tripalmitin 6.67% and tripalmitolein 3.33% (triglycerides), palmitoleic acid 16.67% and palmitic acid 33.3% (fatty acids) | Yes (DSC) | Penetration/drug adsorption |
| **15** | Lu et al. (3) | 2009 | Based on Spangler 1967 | Artificial sebum composition: paraffin wax 15% (wax esters), olive oil 20% and coconut oil 15% (triglycerides), stearic acid 15% and oleic acid 15% (fatty acids), cholesterol 20% | Yes (DSC) | Penetration/drug adsorption |
| **16** | Lu et al. (4) | 2009 | Based on Friberg and Osborne 1986 | Artificial sebum composition: squalene 15%, palmitic acid pamitic ester 10% and oleic acid palmitic ester 10% (wax esters), tripalmitin 20% and triolein 20% (triglycerides), oleic acid 6%, palmitic acid 10% and myristic acid 4% (fatty acids), cholesterol 3%, cholesterol oleate 1%, cholesteryl palmitate 1% (cholesterol esters) | Yes (DSC) | Penetration/drug adsorption |
| **17** | Motwani *et al.* | 2002 | Based on Nordstrom 1986 | Artificial sebum composiiton: squalene 13%, palmitic acid myristyl ester 27% (wax ester), tripalmitin 6.67% and tripalmitolein 3.33% (triglycerides), palmitoleic acid 16.67% and palmitic acid 33.3% (fatty acids) | Yes (DSC) | Penetration/drug adsorption |
| **18** | Gerhard *et al.* | 2009 | Based on Stefaniak 2006 | Artificial sebum composition: triglycerides: tristearin 21.4%, triolein 10.7%, wax esters: palmityl palmitate 19.4%, oleyl oleate 4.8%, free fatty acids: stearic acid 13.8%, oleic acid 13.8%, cholesteryl oleate 1.9%, cholesterol 3.9% and squalene 10.3% | Yes (CoF and Contact angle) | Study of sebum |
| **19** | Wertz | 2009 | New | Artificial sebum composition: 12.4% squalene, 25% wax monoester (jojoba oil), 44.7% triglyceride (triolein), 17% fatty acid (oleic acid) and 0.1% vitamin E | Yes (TLC) | Study of sebum |
| **20** | Mainkar and Jolly | 2000 | New | Artificial sebum composition : triglycerides: olive oil 20%, coconut oil 15%, fatty acids: stearic acid 15%, oleic acid 15%, wax esters: paraffin wax 15%, cholesterol 20% | No | Cleaning/removal |
| **21** | Nelson *et al.* | 1993 | New | Artificial sebum composition: squalene 8%, tristearin 23% and triolein 23% (triglycerides), stearic acid 15% and oleic acid 15% (fatty acids), cholesterol 8%, octadecanol 8% | No | Penetration/drug adsorption |
| **22** | Thompson *et al.* | 1985 | Based on Spangler 1965 | Artificial sebum composition: squalene 5%, triglycerides: olive oil 20%, cocnut oil 15%, fatty acids: palmitic acid 10%, stearic acid 5%, oleic acid 15%, wax esters: paraffin wax 15%, spermaceti 15%, cholesterol 5% | No | Cleaning/removal |
| **23** | Spangler *et al.* | 1965 | New | Artificial sebum composition: squalene 5%, triglycerides: olive oil 20%, cocnut oil 15%, fatty acids: palmitic acid 10%, stearic acid 5%, oleic acid 10%, linoleic acid 5%, wax esters: paraffin wax 10%, spermaceti 15%, cholesterol 5% | No | Cleaning/removal |
| **24** | Hatamleh and Watts | 2010 | Based on Mohite 1994 and Polyzois 2000 | Artificial sebum composition: fatty acids: palmitic acid 10%, linoleic acid 88%, wax esters: glyceryl tripalmitate 2% | No | Penetration/drug adsorption |
| **25** | Stefaniak *et al.* | 2010 | Based on Musial and Kubis | Artificial sebum composition: triglycerides: tristearin 21.4%, triolein 10.7%, wax esters: palmityl palmitate 19.4%, oleyl oleate 4.8%, free fatty acids: stearic acid 13.8%, oleic acid 13.8%, cholesteryl oleate 1.9%, cholesterol 3.9% and squalene 10.3% | Yes (TLC) | Study of sebum |
| **26** | Komesvarakul *et al.* | 2006 | Prepared by Mary Kay Inc and liquid at 25C | Artificial sebum composition: fatty acids: lauric acid 11.73%, oleic acid 11.73%, isostearic acid 5.86%, triglycerides: tricaprin 11.73%, triolein 11.73%, wax estres: glycerol triisostearate 5.86%, oleyl oleate 10.6%, myristyl myristate 10.6%, isostearyl isostearate 4.13%, squalene 12.23, cholesterol 1.53%, cholesterol oleate 2.27% | No | Cosmetic/dermatology |
| **27** | Hatamleh *et al.* | 2011 | Based onMohite 1994 and Polyzois 2000 | Artificial sebum composition: fatty acids: palmitic acid 10%, linoleic acid 88%, wax esters: glyceryl tripalmitate 2% | No | Penetration/drug adsorption |
| **28** | Hatamleh and Watts | 2011 | refers to Mohite 1994 and Polyzois 2000 | Artificial sebum composition: fatty acids: palmitic acid 10%, linoleic acid 88%, wax esters: glyceryl tripalmitate 2% | No | Penetration/drug adsorption |
| **29** | Sakamoto *et al.* | 2012 | Based on Nordstrom 1986 | Artificial sebum composition: 4% cholesterol, 25% palmitic acid stearyl ester (wax ester), 20% squalene, 16% tripalmitin (triglyceride), 33% palmitic acid (fatty acid) | Yes (Absorption) | Cosmetic/dermatology |
| **30** | Yokoi  *et al.* | 2014 | New | Artificial sebum composition: squalene 9%, myristyl myristate 24% (wax ester), triglyceride 47%, cholesterol 2%, cholesterol ester 2%, free fatty acid 15%, carbon black 2% | No | Cleaning/removal |
| **31** | Jones *et al.* | 2016 | WFK sebum | Artificial sebum composition: hydrocarbon mixture 12%, wax esters: lanoline 18.3%, cutina 11.6%, triglycerides: beef tallow 32.8%, fatty acid triglycerides 3.6%, fatty acids: free fatty acids 18% , cholesterol 3.7% | No | Cleaning/removal |
| **32** | Nandy *et al.* | 2016 | Scientific services | Artificial sebum composition. Wax esters: paraffin oil, artificial spermaceti wax, triglycerides: coconut oil, oilive oil, cholesterol, fatty acids: distilled tallow fatty acids | No | Cosmetic/dermatology |
| **33** | Parker and Morrison (1) | 2016 | Based on Lu 2009 and Stefaniak 2010 , with fatty acids | Artificial sebum composition: squalene 10.6%, wax esters: paraffin wax 10%, spermaceti 15%, triglycerides: olive oil 7%, coconut oil 7%, cottonseed oil 17%, fatty acids: oleic acid 13.8%, steric acid 13.8%, free cholesterol 3.9%, cholesterol oleate 1.9%, vitamin E trace | No | Penetration/drug adsorption |
| **34** | Parker and Morrison (2) | 2016 | Based on Lu 2009 and Stefaniak 2010 , without fatty acids | Artificial sebum composition: squalene 14.6%, wax esters: paraffin wax 13.8%, spermaceti 20.7%, triglycerides: olive oil 9.7%, coconut oil 9.7%, cottonseed oil 23.4%, fatty acids: oleic acid 0%, steric acid 0%, free cholesterol 5.4%, cholesterol oleate 2.6%, vitamin E trace | No | Penetration/drug adsorption |
| **35** | Schneider et al. | 2016 | Based on Stefaniak 2006 | Artificial sebum composition: squalene 10.4%, wax esters: lanolin 24.4%, triglycerides: tripalmitin 15.9%, tristearin 15.9%, fatty acids: oleic acid 13.8%, palmitic acid 6.9%, stearic acid 6.9%, cholesteryl oleate 2.0%, cholesterol 3.9% | No | Penetration/drug adsorption |
| **36** | Stoehr *et al.* | 2016 | WFK sebum | Artificial sebum composition: hydrocarbon mixture 12%, wax esters: lanoline 18.3%, cutina 11.6%, triglycerides: beef tallow 32.8%, fatty acid triglycerides 3.6%, fatty acids: free fatty acids 18% , cholesterol 3.7% | Yes (SEM, Rheology) | Fingerprint study |
| **37** | Baalbaki and Kasting (RN289) | 2017 | Based on Lu 2009 | Artificial sebum composition: squalene 15%, Paraffin wax 10% (wax ester), spermaceti wax (wax ester) 15%, olive oil (triglycerides) 10, cotton seed oil (triglycerides) 25%, coconut oil (triglycerides) 10%, fatty acids: oleic acid (1.4%), palmitic acid (5%), palmitoleic acid (5%), cholesterol (1.2%), cholesterol oleate (2.4%) | No | Penetration/drug adsorption |
| **38** | Baalbaki and Kasting (RN292) | 2017 | Based on Lu 2009 | Artificial sebum composition: squalene 15%, Paraffin wax 10% (wax ester), spermaceti wax (wax ester) 15%, olive oil (triglycerides) 10, cotton seed oil (triglycerides) 25%, coconut oil (triglycerides) 10%, fatty acids: oleic acid (1.4%), palmitic acid (5%), palmitoleic acid (5%), cholesterol (1.2%), cholesterol oleate (2.4%) | No | Penetration/drug adsorption |
| **39** | Galliano *et al.* | 2017 | New | Oleic acid 20%v/v and 80% v/v thermal water | Yes (CoF) | Cosmetic/dermatology |
| **40** | Lawrence *et al.* | 2017 | Scientific services | Artificial sebum composition (no weight % given). Wax esters: paraffin oil, artificial spermaceti wax, triglycerides: coconut oil, oilive oil, cholesterol, fatty acids: distilled tallow fatty acids | No | Cleaning/removal |
| **41** | Pawar *et al.* | 2017 | Based on Stefaniak 2006, Stefaniak 2008 (patent) | Artificial sebum composiiton: squalene 24.3%, wax esters: palmityl palmitate 45.9%, triglycerides: triolein 25.2%, cholesteryl oleate 4.6% | No | Cosmetic/dermatology |
| **42** | Peterson et al. | 2017 | New | Artificial sebum composition: squalene 7%, wax esters: polyglyceryl oleate 2.5%, glyceryl trioleate 15%, cetyl palmitate 8%, mineral oil 10%, triglycerides: petrolatum 10%, fatty acids: oleic acid 15%, cholesterol 3.5%, other: water 20%, carbon black 4%, iron oxides 4%, preservative 1% | No | Cleaning/removal |
| **43** | Doran and Howitt | 2019 | Pickering Laboratories artificial sebum | Aritifical sebum composition: wax esters: synthetic spermacetti 15-35%, paraffin waxes 5-10%, triglycerides: virgin olive oil 15-35%, fractionated coconut oil 15-35%, fatty acids: palmitic acid 5-10%, linoleic acid 2-12%, oleic acid 5-10%, stearic acid 2-12%, cholesterol 2-12, 2,6,10,15,19,23-hexamethyltetracosa-2,6,10,14,18,22-hexaene 2-12% | No | Penetration/drug adsorption |
| **44** | Druart *et al.* | 2018 | Based on Wertz 2009 | Artificial sebum composition: 12.4% squalene, 25% wax monoester (jojoba oil), 44.7% triglyceride (triolein), 17% fatty acid (oleic acid) and 1% vitamin E | No | Fingerprint study |
| **45** | Spittaels and Coenye | 2018 | New | Artificial sebum composition: squalene 15.4%, wax esters: jojoba oil 25%, triglycerides: tripalmitin 41%, triolein 10.3%, fatty acids: palmitic acid 6.2%, cholesterol 2.1%, other: tocopherol acetate 0.01% | No | Cosmetic/dermatology |
| **46** | Yang *et al.* | 2018 | Based on Valiveti 2007, 2008 | Artificial sebum composition: squalene 15%, Paraffin wax 10% (wax ester), spermaceti wax (wax ester) 15%, olive oil (triglycerides) 10, cotton seed oil (triglycerides) 25%, coconut oil (triglycerides) 10%, fatty acids: oleic acid (1.4%), palmitic acid (5%), palmitoleic acid (5%), cholesterol (1.2%), cholesterol oleate (2.4%) | No | Penetration/drug adsorption |
| **47** | Borrel *et al.* | 2019 | Mention Catroux 2005 (expired patent), Stefaniak 2010, Wertz 2009 but not follow the formulas | Artificial sebum composition: squalene 12.6%, triolein 45%, oleic acid 17.2%, jojoba oil 25.2% | No | Cosmetic/dermatology |
| **48** | Villegas *et al.* (1) | 2019 | Based on Stefaniak 2010 | Artificial sebum composition: triglycerides: tristearin 21.79%, triolein 10.9%, wax esters: lanolin 24.75%, free fatty acids: palmitic acid 14.05%, oleic acid 14.05%, cholesterol 3.97% and squalene 10.49% | No | Penetration/drug adsorption |
| **49** | Villegas et al. (2) | 2019 | Based on Wertz 2009 | Artificial sebum composition: 12.4% squalene, 25% wax monoester (jojoba oil), 44.7% triglyceride (triolein), 17% fatty acid (oleic acid) and 1% vitamin E | No | Penetration/drug adsorption |
| **50** | Yang *et al.* | 2019 | Based on Valiveti 2007, 2008 | Artificial sebum composition: squalene 15%, Paraffin wax 10% (wax ester), spermaceti wax (wax ester) 15%, olive oil (triglycerides) 10, cotton seed oil (triglycerides) 25%, coconut oil (triglycerides) 10%, fatty acids: oleic acid (1.4%), palmitic acid (5%), palmitoleic acid (5%), cholesterol (1.2%), cholesterol oleate (2.4%) | No | Penetration/drug adsorption |
| **51** | Antunes and Cavaco-Paolo | 2020 | Based on Lu 2009, Stefaniak 2010, Nordstrom | Sebum used for modelling (not physical sebum): squalene 10%, wax esters: palmityl palmitate 14%, oleyl oleate 12%, triglycerides: tripalmitin 20%, triolein 12%, fatty acids: palmitic acid 12%, palmitoleic acid 15%, cholesterol 2%, cholesterol oleate 4% | Yes (Molecular Dynamics) | Penetration/drug adsorption |
| **52** | Kim *et al.* | 2022 | Based on Wertz 2009 | Artificial sebum composition: squalene 12.5%,wax ester: jojoba oil 25.2%, triglycerides 45.1%, fatty acids: oleic acid 17.2% | No | Penetration/drug adsorption |
| **53** | Eudier *et al.* | 2020 | Based on Stefaniak 2010 | Artificial sebum composition: squalene 10.29%, wax esters: palmityl palmitate 19.42%, oleayl olaeta 4.85%, triglycerides: tristearin 21.36%, triolein 10.68%, fatty acids: stearic acid 6.87%, palmitic acid 6.87%, oleic acid 13.74, colesteryl oleate 1.94%, cholesterol 3.88, alpha tocopherol 0.1% | Yes (Imaging, Contact Angle, IR) | Cosmetic/dermatology |
| **54** | Korbeld *et al.* | 2020 | Based on Lu 2009 | 5 formulations used, all of them had varying amounts of: squalene, palmitic acid, glyceryl trioleate, paraffin, cholesterol. No cholesterol esters used as in normal sebum present in trace amount. Squalene 12.4-20.3%, Wax 19.3-27.0%, Triglyceride 16.4-65.0%, Fatty acids 0 -33.6%, Cholesterol 0.6-3.9% | Yes (CoF) | Study of sebum |
| **55** | Miracle *et al.* | 2020 | Accurate Product Development artificial body soil (ABS) | Artificial sebum composition: squalene 5%, wax esters: paraffin 15%, triglycerides: coconut oil 15%, olive oil 15%, cottonseed oil 15%, fatty acids: steric acid 5%, palmitic acid 5%, oleic acid 15%, myristic acid 5%, cholesterol 5% | No | Cleaning/removal |
| **56** | Puccetti and Kulcsar | 2020 | Scientific services | Artificial sebum composition: squalene 1-10%, wax esters: paraffin wax 5-20%, spermacetti 8-20%, triglycerides: coconut oil 10-22%, olive oil 10-30%, fatty acids: palmitic acid 5-25%, stearic acid 2-15%, oleic acid 4-15%, linoleic acid 2-13%, cholesterol 2-13% | No | Cosmetic/dermatology |
| **57** | Gorka *et al.* | 2021 | Based on Sisco 2015 | Artificial sebum composition: squalene 8.6%, wax esters: cetyl palmitate 11.2%, triglycerides: triolein 19.8%, tricaprylin 1.4%, tricaprin 1.4%, trilaurin 1.4%, trimyristin 1.4%, tripalmitin 1.4%, fatty acids: (3.6% of each of: hexanoic acid, hepttanoic acid, octanoic acid, nonanoic acid, dodecanoic acid, tridecanoic acid, pentadecanoic acid, arachidic acid) and 4% of each of palmitic acid, stearic acid, linoleic acid, oleic acid, cholesterol n-docanoate 2.9%, cholesterol 2.2% | No | Cleaning/removal |
| **58** | Stefaniak *et al.* | 2021 | Based on Musial and Kubis | Artificial sebum composition: triglycerides: tristearin 21.4%, triolein 10.7%, wax esters: palmityl palmitate 19.4%, oleyl oleate 4.8%, free fatty acids: stearic acid 13.8%, oleic acid 13.8%, cholesteryl oleate 1.9%, cholesterol 3.9% and squalene 10.3% | No | Cosmetic/dermatology |
| **59** | Champmartin *et al.* | 2022 | Based on Downing 1969, Greene 1970, Stefaniak 2006, Valiveti 2007, Lu 2009, Tsai 2012 | This is average composition of artifical sebums. Squelene 15%, wax esters 24%, triglycerides 34%, free fatty acids 22%, cholesterol 2.1%, cholesterol esters 2.4%, diglycerides 0.6% | No | Penetration/drug adsorption |
| **60** | Champmartin *et al.* | 2020 | Based on Stefaniak 2006, Valiveti 2007, Lu 2009, Wertz 2009, Stefaniak 2010 | Artificial sebum composition: squalene 15%, wax esters:jojoba oil 25% , triglycerides: glycerol trioleate 30%, fatty acids: oleic acid 30% | No | Penetration/drug adsorption |
| **61** | K. Suzuki *et al.* | 2022 | New | Artificial sebum composition: squalene 11.3%, triglycerides: triolein 58.6%, fatty acids: palmitic acid 6.1%, myristic acid 2.2%, pentadecanoic acid 1.5%, oleic acid 4.7%, other: glyceryl distearate 15.6% (diglyceride) | No | Cosmetic/dermatology |
| **62** | Galliano *et al.* | 2023 | Based on Peterson 2017 | Artificial sebum composition: squalene 7%, wax esters: polyglyceryl oleate 2.5%, glyceryl trioleate 15%, cetyl palmitate 8%, mineral oil 10%, triglycerides: petrolatum 10%, fatty acids: oleic acid 15%, cholesterol 3.5%, other: water 20%, carbon black 4%, iron oxides 4%, preservative 1% | No | Cleaning/removal |
| **63** | Kostrzebska *et al.* | 2023 | Based on Musial and Kubis | Artificial sebum composition: squalene 12%, wax esters: lanolin 26%, triglycerides: pork lard 34%, fatty acid: stearic acid 24%, cholesterol 4% | No | Cosmetic/dermatology |
| **64** | Swaney *et al.* | 2023 | Based on Lu 2009 , sebum L | Artificial sebum composition: squalene 15%, wax esters: paraffin wax 10%, hexadecyl palmitate 15%, triglycerides: olive oil 10%, coconut oil 10%, cottonseed oil 25%, fatty acids: oleic acid 1.4%, myristic acid 2.5%, lauric acid 2.5%, palmitic acid 5%, cholesterol oleate 2.4%, cholesterol 1.2% | No | Cosmetic/dermatology |
| **65** | Arsenault *et al.* | 2023 | Based on Sisco 2015 | Artificial sebum composition: squalene 9%, wax esters: cetyl palmitate 11.6%, triglycerides: triolein 20.6%, tricaprylin 1.5%, tricaprin 1.5%, trilaurin 1.5%, trimyristin 1.5%, tripalmitin 1.5%, fatty acids: (3.75% of each of: hexanoic acid, heptanoic acid, octanoic acid, nonanoic acid, dodecanoic acid, tridecanoic acid, myristic acid, pentadecanoic acid, arachidic acid) and 4.12% of each of palmitic acid, stearic acid, linoleic acid, cholesterol n-docanoate 3%, cholesterol 2.2% | No | Fingerprint study |
| **66** | De La Hunty | 2017 | New | Artificial sebum composition: squalene 13.9%, triglycerides: vegetable oil 41.7%, fatty acids: stearic acid 8.7%, palmitic acid 8.7%, oleic acid 18.3%, stigmasterol (cholesterol ester) 2.6%, cholesterol 5.2%, Vitamin E oil (alpha-tocopherol) 0.9% | No | Fingerprint study |
| **67** | Sisco *et al.* | 2015 | New | Artificial sebum composition: squalene 8.6%, wax esters: cetyl palmitate 11.2%, triglycerides: triolein 19.8%, tricaprylin 1.4%, tricaprin 1.4%, trilaurin 1.4%, trimyristin 1.4%, tripalmitin 1.4%, fatty acids: (3.6% of each of: hexanoic acid, hepttanoic acid, octanoic acid, nonanoic acid, dodecanoic acid, tridecanoic acid, pentadecanoic acid, arachidic acid) and 4% of each of palmitic acid, stearic acid, linoleic acid, oleic acid, cholesterol n-docanoate 2.9%, cholesterol 2.2% | Yes (SIMS) | Fingerprint study |
| **68** | Steiner *et al.* (1) | 2022 | Based on De la Hunty 2017 | Artificial sebum composition: squalene 13.9%, triglycerides: vegetable oil 41.7%, fatty acids: stearic acid 8.7%, palmitic acid 8.7%, oleic acid 18.3%, stigmasterol (cholesterol ester) 2.6%, cholesterol 5.2%, Vitamin E oil (alpha-tocopherol) 0.9% | No | Fingerprint study |
| **69** | Steiner et al. (2) | 2022 | Based on Sisco 2015 | Artificial sebum composition: squalene 8.6%, wax esters: cetyl palmitate 11.2%, triglycerides: triolein 19.8%, tricaprylin 1.4%, tricaprin 1.4%, trilaurin 1.4%, trimyristin 1.4%, tripalmitin 1.4%, fatty acids: (3.6% of each of: hexanoic acid, hepttanoic acid, octanoic acid, nonanoic acid, dodecanoic acid, tridecanoic acid, pentadecanoic acid, arachidic acid) and 4% of each of palmitic acid, stearic acid, linoleic acid, oleic acid, cholesterol n-docanoate 2.9%, cholesterol 2.2% | No | Fingerprint study |
| **70** | Bajagain *et al.* | 2023 | Based on Kim 2022, Stefaniak 2010, Wertz 2009 | Artificial sebum composition: squalene 12.4%, WE: jojoba oil 25%, TR: triolein 44.6%, FA: oleic acid 17%, Vitamin E +/- alpha tocopherol 1% | No | Penetration/drug adsorption |
| **71** | Abdel-Mottaleb *et al.* | 2015 | Based on Lu 2009 | Artificial sebum composition: squalene 15%, Paraffin wax 10% (wax ester), spermaceti wax (wax ester) 15%, olive oil (triglycerides) 10, cotton seed oil (triglycerides) 25%, coconut oil (triglycerides) 10%, fatty acids: oleic acid (1.4%), palmitic acid (5%), palmitoleic acid (5%), cholesterol (1.2%), cholesterol oleate (2.4%) | No | Penetration/drug adsorption |
| **72** | Kattou et al. | 2017 | Based on Valiveti 2007, 2008 | Artificial sebum composition: squalene 15%, Paraffin wax 10% (wax ester), spermaceti wax (wax ester) 15%, olive oil (triglycerides) 10, cotton seed oil (triglycerides) 25%, coconut oil (triglycerides) 10%, fatty acids: oleic acid (1.4%), palmitic acid (5%), palmitoleic acid (5%), cholesterol (1.2%), cholesterol oleate (2.4%) | No | Penetration/drug adsorption |
| **73** | Lauterbach and Mueller-Goymann | 2014 | Based on Lu 2009 | Artificial sebum composition: squalene 15%, wax esters: cetyl palmitate 20%, oleyl oleate 5%, triglycerides: olive oil 35%, coconut oil 12%, fatty acids: oleic acid 5%, palmitic acid 5%, cholesterol 1%, cholesterol oleate 2% | No | Penetration/drug adsorption |
| **74** | Mart'yanov *et al.* | 2022 | Based on Spittaels and Coenye 2018 | Artificial sebum composition: 54% glycerol tripalmitate (triglyceride), 46% jojoba oil (wax esters) | No | Cosmetic/dermatology |
| **75** | Pannu e*t al.* | 2011 | Based on Lu 2009 | Artificial sebum composition: squalene 15%, wax esters: cetyl palmitate 20%, oleyl oleate 5%, triglycerides: olive oil 35%, coconut oil 12%, fatty acids: oleic acid 5%, palmitic acid 5%, cholesterol 1%, cholesterol oleate 2% | No | Cosmetic/dermatology |
| **76** | Reifenrath *et al.* | 2023 | Pickering Laboratories artificial sebum (Pickering Laboratories manufactures an artificial sebum formulation according to ASTM designation D4265-14 or D4265-98. ) | n/a | No | Penetration/drug adsorption |
| **77** | Staymates *et al.* | 2013 | Based on Valiveti 2008, Musial and Kubis 2003, Airey 2006 (ASTMD4265-98 standard) | Artificial sebum composition: squalene 5%, wax esters: paraffin wax 10%, jojoba oil 15%, triglycerides: olive oil 20%, coconut oil 15%, fatty acids: palmitic acid 10%, stearic acid 5%, oleic acid 10%, linoleic acid 5%, cholesterol 5% | No | Fingerprint study |
| **78** | Stefaniak *et al.* | 2011 | Based on Stefaniak 2010 | Artificial sebum composition: triglycerides: tristearin 21.4%, triolein 10.7%, wax esters: palmityl palmitate 19.4%, oleyl oleate 4.8%, free fatty acids: stearic acid 13.8%, oleic acid 13.8%, cholesteryl oleate 1.9%, cholesterol 3.9% and squalene 10.3% | No | Penetration/drug adsorption |
| **79** | Runnsjo *et al.* | 2022 | no information | Artificial sebum composition: olive oil, coconut oil, cottoseed oil and squalane. No information on the amounts given | No | Cosmetic/dermatology |
| **80** | Friberg and Osborne | 1986 | Based on Downing 1969, Haahti 1961, O'Neill 1969 | Artificial sebum composition: squalene 12.2%, WE: oleic acid palmytic ester 20.3%, TR: triolein 41.8%, FA: oleic acid 16.5% myristic acid 1.9%, CHE: cholesteryl oleate 3%, other: pristane 2.8%, lecithin 1.5% | No | Cosmetic/dermatology |
| **81** | Blanc *et al.* | 1989 | New | Artificial sebum composition: TR: triglycerides 34%, WE: glycerol isostearate 3%, SQ: squalene 15%, lanoline 5%, CHE: cholesterol oleate 6%, FA: 37% (lauric 1%, myristic 3%, palmitic 5%, stearic 3%, oleic 25%) | No | Cosmetic/dermatology |
